# The relationship between family communication networks and elderly loneliness: A structural equation modeling approach across three generations

**DOI:** 10.1371/journal.pone.0358707

**Published:** 2026-10-05

**Authors:** Mahdi Sepidarkish, Afsaneh Bakhtiari, Zeinab Baheshmat, Shabnam Omidvar

**Affiliations:** 1 Cellular and Molecular Biology Research Center, Health Research Institute, Babol University of Medical Sciences, Babol, Iran; 2 Social Determinants of Health Research Center, Health Research Institute, Babol University of Medical Sciences, Babol, Iran; 3 Student Research Committee, Babol University of Medical Sciences, Babol, Iran; Tung Wah College, HONG KONG

## Abstract

**Background and Objective:**

Loneliness is a widespread challenge among older adults, often linked to negative health outcomes. This study explores how family communications among individual members representing three generations is associated with loneliness in elderly participants, aiming to clarify which family relationships are most impactful.

**Methods:**

Using partial least squares structural equation modeling, we examined the effects of marital intimacy in both the older generation (G1) and their adult children (G2), as well as broader family factors such as intergenerational solidarity, family cohesion, sibling relationship, in-law interaction, children’s support for parents and grandparent-grandchild relationship, on loneliness in elderly family members.

**Results:**

The findings revealed that marital intimacy within the G2 was the only direct predictor associated with reduced loneliness among older adults (β = −0.314, p = 0.019). The marital closeness of the older generation showed only a weak association (β = −0.194, p = 0.067). Other family relationship factors, including general cohesion and intergenerational bonds, did not demonstrate meaningful direct or indirect effects. Importantly, the quality of G1 marital relationships showed indirect associations with loneliness by strengthening the marital bonds of G2, highlighting a pattern of relationship qualities being passed between generations.

**Conclusion:**

This study suggests that it is not broad family connectedness but rather the quality of key dyadic relationships, especially among middle-aged couples, that matters most for elderly loneliness. Efforts to enhance marital relationships in the middle generation may be associated with lower loneliness for their aging parents.

## Introduction

Loneliness among older adults has emerged as a pressing public health concern worldwide, given its well-established links to adverse physical and mental health outcomes, including increased risk of depression, cognitive decline, and mortality [1,2]. Traditionally, the scholarly focus on mitigating loneliness in elderly populations has centered on broad constructs such as family cohesion and generalized social support networks, which emphasize the overall size of the family network, frequency of contact, or perceived availability of assistance [3,4]. This conventional perspective largely treats family connectedness as a quantitative phenomenon, assuming that higher levels of contact and broader support are associated with better emotional well-being [5]. Not all family relationships carry the same emotional weight, and certain dyadic ties may be more consequential than others for elderly loneliness.

Recent theoretical developments support this distinction [6]. The Convoy Model of social relations posits that social ties vary in function and emotional quality across the lifespan, suggesting that emotionally rich dyadic relationships may be more influential than broad social integration [7]. One particularly relevant dyad is the marital relationship within the middle generation. Adults in their 40s and 50s, often referred to as the “pivot generation,” occupy a unique structural position in the family. They typically coordinate family gatherings, mediate intergenerational conflicts, and serve as the primary conduit for emotional and practical support to aging parents [8]. When their own marriage is characterized by closeness and mutual support, they tend to perform these functions more effectively. Conversely, marital distress among middle-aged couples may disrupt the entire family support system. Empirical evidence indicates that marital intimacy within this generation correlates with relational health across multiple family members [9,10]. Furthermore, research on intergenerational transmission demonstrates that parental marital quality shapes offspring’s relationship dynamics, suggesting potential indirect pathways across generations [11].

However, focusing exclusively on the middle generation’s marriage provides an incomplete picture. Older adults are embedded within a broader network of family relationships spanning three generations, including their own marital bond (G1), relationships with adult children and their spouses, ties with grandchildren, and in some cases sibling connections. The term “family communication network” here refers to the patterns of emotional exchange across these various dyadic relationships, as opposed to a simple count of social contacts. How these diverse connections collectively are related to loneliness remains unclear from existing literature. Some studies report protective effects of general family cohesion, whereas others emphasize specific intimate bonds as primary determinants of emotional well-being in later life [12–14]. Resolving this ambiguity matters because without it, interventions to reduce elder loneliness may target the wrong family relationships.

The present study addresses these gaps by applying structural equation modeling to examine how family communication networks across three generations are associated with loneliness in older adults. Rather than treating the family as a single source of support, we disaggregate the network into specific dyadic relationships: marital intimacy in the older generation (G1) and the middle generation (G2), parent-child support, grandparent-grandchild bonds, sibling relationships, in-law interactions, and general family cohesion. This design allows us to determine whether broad family connectedness or specific dyadic are associated with elderly loneliness, and whether any effects operate directly or indirectly across generations. By doing this in the Iranian family context, we offer a framework that other researchers can replicate with their own samples in different cultural settings. Based on family systems and intergenerational relationship theories, we expected that stronger dyadic and broader family relational ties would be associated with lower loneliness among older adults. Specifically, we hypothesized that: (1) higher marital intimacy in both G1 and G2 would be associated with lower loneliness; (2) broader family relational factors, including cohesion, intergenerational solidarity, children’s support, and grandparent-grandchild ties, would be related to loneliness; and (3) these associations might partly operate through indirect intergenerational pathways.

## Methods

### Design study

This community-based cross-sectional study was conducted between 15^th^ September 2022 and 10^th^ March 2023 in Jouybar, a city in the northern part of Iran, Mazandaran province. Iran’s relatively strong intergenerational family ties make it a relevant context for examining family relational pathways across generations. The research aimed to assess the relationship between family communication networks and psychosocial well-being, specifically loneliness and social isolation, among older adults. The analysis encompassed a multi-generational perspective by examining family communication across three generations: first generation-G1 (older adults/grandparents), second generation-G2 (adult children), and third generation-G3 (grandchildren). This approach provided insights into intergenerational connections at the individual family level, though findings should not be generalized to the entire societal generation. According to recent municipal data, Jouybar has a population of around 34,000, with an estimated 4,050 older adults, representing 11.9% of the total population. The city is served by three government-run Comprehensive Health Service Centers, which maintain up-to-date health records for all households within their designated coverage zones.

### Participants

The study enrolled married couples aged 60 years or older who were permanent residents of Jouybar. To be eligible, participants had to meet the following criteria: (1) no prior remarriage, (2) at least two married children with grandchildren, (3) regular access to children and grandchildren (either in person or via telephone), (4) functional independence (no major limitations in basic or instrumental activities of daily living), (5) absence of significant hearing, vision, or speech impairments that could interfere with communication, and (6) no terminal illness, recent severe acute disease (within the last three months), or current use of medications for Alzheimer’s disease or depression. Individuals were excluded if: (1) their children or grandchildren lived abroad, (2) they had severe cognitive decline or required extensive assistance with daily activities, or (3) they refused participation upon initial contact.

### Sampling strategy and data collection

Each sampling unit consisted of a three-generation family, including an older couple, one adult child with their spouse, and one grandchild. To achieve a representative sample of elderly couples in Jouybar, a two-stage sampling approach was used. In the first stage, stratified sampling was applied using the city’s three Comprehensive Urban Health Service Centers, which represent all such centers in Jouybar, as separate strata. This ensured appropriate geographical coverage. In the second stage, simple random sampling was conducted within each stratum. Eligible couples were identified from health center records and selected using Stata’s computerized random number generator to reduce selection bias.

Eligible older adults were invited to attend the health centers. At the visit, the study objectives and procedures were explained, and written informed consent was obtained. The questionnaires were then administered. Questionnaires for the first generation (older adults) were completed through face-to-face interviews by a trained researcher. For the second and third generations, the study was introduced during the initial telephone contact. Verbal informed consent was obtained before starting the interview, and consent was audio-recorded by the researcher. Questionnaires were then completed via telephone interview by the same researcher.

### Sample size calculation

The required sample size was determined based on the study’s primary objective of examining the association between family communication networks and loneliness. A minimum correlation coefficient of 0.30 was used as the reference effect size, derived from prior studies on loneliness and family networks in similar populations [15]. Using PASS software (version 21) [16], with a two-tailed test, 95% confidence level (α = 0.05), and 80% power (β = 0.20), the initial calculation yielded a requirement of 85 elderly couples (170 individuals). To account for a potential 10% non-response rate or incomplete data, the target sample size was adjusted upward to 94 couples (188 individuals). In practice, we enrolled 107 eligible couples who met all inclusion criteria. Following the exclusion of five cases due to incomplete data, the final analytic sample comprised 107 older adults (representing 107 couples). Alongside the first-generation participants, 107 adult children, their 107 spouses, and 107 grandchildren were also enrolled, resulting in a total of 428 individuals representing three generational levels within extended families.

### Measurements

The study used established instruments and Iranian-developed measures with previously reported psychometric evidence. To complement the existing psychometric evidence, we further assessed construct validity via confirmatory factor analysis (CFA) using AMOS version 24. A brief synopsis of the psychometric properties is presented below, whereas the comprehensive validation evidence is set out in، Table 2 and S1 Table.

### Sociodemographic and health characteristics questionnaire

The sociodemographic questionnaire systematically captured key participant characteristics across three domains. Demographic information included age, sex, occupational status, educational attainment, and marital status. Living arrangements were categorized as living alone, cohabiting with a spouse, residing with both spouse and unmarried children, or other household configurations. Economic status was assessed through participant self-reporting. Health-related variables encompassed current medication use (with polypharmacy operationally defined as consumption of four or more daily medications), physician-diagnosed chronic conditions (including cardiovascular, metabolic, endocrine, respiratory, musculoskeletal and renal diseases), hospitalization history within the preceding year, and current tobacco use status. This comprehensive assessment enabled detailed characterization of the study population’s baseline profile.

### De Jong Gierveld loneliness scale

The De Jong Gierveld Loneliness Scale (1985) measures loneliness through emotional (6 items) and social (5 items) components. The Persian version validated by Hosseini et al. (2021) [17] for Iranian older adults omitted three items, leaving 8 items (5 social, 3 emotional). Following standard scoring, each response is recoded as 0 or 1, yielding a total score ranging from 0 to 8, with higher scores indicating greater loneliness. Example item: “There are enough people I feel close to.” The translation followed forward-backward procedures with expert committee review. CFA confirmed a two-factor structure with acceptable fit (CFI = 0.92, RMSEA = 0.06). Construct validity was supported by significant correlations with depression (r = 0.48) and perceived social support (r = −0.41), while known-groups validity demonstrated significant differences by living arrangement (p < 0.001). The scale showed good internal consistency in the Iranian population (Cronbach’s α > 0.80) [17].

### Family communication network questionnaires

To identify types of intergenerational relationships in the elderly family, the classification framework proposed by Silverstein et al. [18] was employed.

### First generation (elderly couples)

#### Marital intimacy scale.

The 17-item marital intimacy scale, originally conceptualized by Thompson and Walker (1983), assesses relationship closeness using a 7-point Likert response format (1 = “never” to 7 = “always”).Total scores range from 17 to 119, with higher values reflecting stronger perceived intimacy. The Persian version validated by Salehi Moghadam et al. (2024) [19] underwent forward-backward translation with expert committee review and cognitive debriefing. Exploratory factor analysis (EFA) revealed a single-factor solution accounting for 58.7% of variance, while CFA confirmed the unidimensional structure (CFI = 0.91, RMSEA = 0.07). Construct validity was evidenced by significant positive correlations with marital satisfaction (r = 0.62) and negative correlations with marital conflict (r = −0.55). Known-groups validity was supported by significant differences across educational levels (p < 0.001). The instrument demonstrated exceptional internal consistency (Cronbach’s α = 0.96) and test-retest reliability (ICC = 0.92) in the Iranian population [19].

### Intergenerational solidarity questionnaire

This 16-item multidimensional instrument, based on the framework of Silverstein et al. (2010) [18], assesses three key dimensions of grandparent-grandchild relationships: (1) emotional attachment (rated 1 [“very bad”] to 4 [“very good”]), (2) social attachment (1 [“never”] to 5 [“always”]), and (3) functional attachment (1 [“none”] to 4 [“a lot”]). Raw scores are converted to a standardized 0–100 metric, where elevated scores reflect stronger intergenerational connections. The Persian version by Momtaz et al. (2018) [20] was validated through forward-backward translation and expert panel review. CFA confirmed the three-factor structure with good fit (CFI = 0.921, RMSEA = 0.063), and the scale demonstrated acceptable internal consistency (α = 0.86).

### Second generation (adult children)

#### Marital intimacy questionnaire.

The measure for assessing it, originally conceptualized by Thompson and Walker (1983), has been introduced earlier [19].

### Family cohesion scale

This 28-item instrument evaluates family cohesion across eight distinct dimensions: (1) mother-child bonding, (2) father-child bonding, (3) shared time, (4) interaction settings, (5) emotional connections, (6) joint decision-making, (7) marital dynamics, and (8) parent-child relationships. Items are rated on a 5-point Likert scale (1 = strongly disagree to 5 = strongly agree), yielding total scores between 28–140, with scores ≥96 indicating high family cohesion. The scale was originally developed in Iran by Samani [21]. EFA supported an eight‑subscale structure (inter‑factor r: 0.15–0.73; item‑total r: 0.64–0.79). Convergent and discriminant validity were supported by correlations with the Family Process Scale (r = 0.56, p < 0.001) and Marital Problems Scale (r = –0.24, p < 0.05), respectively, and the scale distinguished divorced from intact families (t = 3.96, p < 0.001). Internal consistency was acceptable (α = 0.79).

### Sibling relationship index

This 25-item instrument [22] evaluates the quality of sibling relationships, with higher scores indicating greater relational difficulties. Items are rated on a 7-point Likert scale (1 = never to 7 = always). The original scale demonstrates strong psychometric properties, including excellent internal consistency (α ≥ 0.90) and established content, construct, factorial, and known-groups validity (Walmyr Publishing Company, 2015). Because no formal psychometric validation of the Persian version was available [23], construct validity was evaluated in the present study using CFA, which supported the proposed one-factor model (S1 Table). Internal consistency was excellent (α = 0.954).

### In-law interaction questionnaire

This 50-item instrument assesses conflict dynamics between married couples and their parents‑in‑law across five domains: interference in marital decisions, boundary violations, communication difficulties, cultural/value conflicts, and financial tensions. Items are rated on a 5‑point Likert scale (1 = strongly disagree to 5 = strongly agree), with total scores ranging from 50 to 250; higher scores indicate greater relational conflict. The questionnaire was developed through qualitative research and expert review. Content validity was established during instrument development, whereas construct validity was further evaluated in the present study using CFA, which supported the proposed five-factor structure. Internal consistency was excellent in both the original study (α = 0.96) and the present study (α = 0.962) [[24]; S1 Table)].

### Children’s support for parents scale

This 7-question instrument assesses adult children’s support from their parents in three dimensions, including instrumental support, financial support, and emotional support. Items are rated on Likert scales tailored to each dimension: instrumental support items range from 0 (never) to 6 (daily), financial support is measured on a 0–6 scale, and emotional support items range from 0 (none) to 5 (very much). The scale was developed in Iran based on items adapted from the NSFH and SHARE surveys [25]. Content validity was established through expert review, EFA supported the original three-factor structure, and CFA in the present study confirmed this structure. Internal consistency was acceptable (α = 0.792) (S1 Table).

### Third generation (grandchildren)

#### Grandparent-grandchild scale.

The grandparent-grandchild Bonding Scale is a 10-item psychometric instrument designed to evaluate the quality of intergenerational relationships across two key dimensions: emotional connectedness and participatory engagement. Utilizing a 4-point Likert response format (1 = “not at all” to 4 = “very much”), the measure yields a total score ranging from 10 to 40, where higher scores reflect stronger relational bonds. The Persian version was validated in a methodological study involving Iranian students. The instrument demonstrated excellent content validity (CVI = 0.98), CFA supported the two-factor structure, and internal consistency was good (α = 0.89) [26].

### Ethical consideration

The Institutional Review Board of Babol University of Medical Sciences granted ethical approval for the study (Approval Ethical Code: IR.MUBABOL.REC.1401.073). This study was conducted under Good Clinical Practice and the principles of the Declaration of Helsinki. Before participation, all individuals provided written informed consent following a detailed explanation of the study’s objectives and procedures.

### Statistical analysis

We conducted our analysis using partial least squares structural equation modeling (PLS-SEM) implemented in SmartPLS 3.0. This multivariate technique was selected for its ability to handle both reflective and formative constructs while making minimal assumptions about data distribution. The analysis proceeded in two sequential stages: measurement model evaluation followed by structural model assessment. For the measurement model evaluation, we examined convergent validity through three established criteria: indicator loadings, average variance extracted (AVE) values, and composite reliability coefficients. We assessed discriminant validity using both the Fornell-Larcker criterion and heterotrait-monotrait (HTMT) ratios. In the structural model assessment, we employed a nonparametric bootstrapping procedure with 5,000 resamples to examine the hypothesized relationships. We calculated path coefficients and their significance levels to test direct effects, while mediation analysis evaluated indirect pathways. The analysis included computation of effect sizes (f^2^) for all significant paths. We assessed the model’s predictive relevance through the blindfolding procedure and evaluated overall model fit using standardized root mean square residual (SRMR) and normed fit index (NFI). To ensure robustness, we conducted supplementary analyses including multi-group analysis of demographic variables and post-hoc power analysis using the inverse square root method. The analysis accounted for both the measurement properties of the constructs and the structural relationships between them, while maintaining appropriate standards for statistical significance and practical relevance.

## Results

### Participant characteristics

Participant demographic and health characteristics are summarized in Table 1. The study enrolled 107 older adult participants with the following demographic and health characteristics. Age distribution revealed a predominance of younger seniors, with 70.1% (n = 75) aged 60–69 years, 25.2% (n = 27) in the 70–79 range, and 4.7% (n = 5) aged 80 or above. The sample included more female participants (58.9%, n = 63) than males (41.1%, n = 44). Educational attainment showed 38.3% (n = 41) had no formal schooling, followed closely by 37.4% (n = 40) with primary education, while secondary (9.3%, n = 10), high school (12.1%, n = 13), and post-secondary (2.8%, n = 3) education were less common. Employment status indicated 71.9% (n = 77) were not currently working, compared to 28.1% (n = 30) who were employed. Health status measures showed 51.4% (n = 55) of participants reported chronic health conditions, with 48.6% (n = 52) indicating no significant medical history. Medication patterns revealed 27.1% (n = 29) met polypharmacy criteria (≥5 medications), while 72.9% (n = 78) did not. These baseline characteristics provide important contextual information about the study population for subsequent analyses.

**Table 1:** Study population characteristics (N =  107).

Variable	Categories	Number	Percent
Age(years)	60–69	75	70.1
	70–79	27	25.2
	80+	5	4.7
Gender	Male	44	41.1
	Female	63	58.9
Education	Illiterate	41	38.3
	Elementary	40	37.4
	Middle	10	9.3
	High	13	12.1
	College or higher	3	2.8
Occupation	Employed	30	28.1
	Unemployed	77	71.9
Underling disease	Yes	55	51.4
	No	52	48.6
Polypharmacy	Yes	29	27.1
	No	78	72.9

### Model fit assessment

The overall fit of the measurement and structural model was evaluated using the standardized root mean square residual (SRMR) and the normed fit index (NFI), which are recommended for PLS-SEM. The SRMR value was 0.072, which is below the commonly accepted threshold of 0.08, indicating an acceptable approximate fit between the hypothesized model and the observed data. The NFI was 0.91, exceeding the recommended cutoff of 0.90, suggesting that the model represents a substantial improvement over a null model. Additionally, the squared Euclidean distance (d_ULS) was 1.234 and the geodesic distance (d_G) was 0.876. For all endogenous constructs, the Stone-Geisser Q^2^ values (obtained via the blindfolding procedure) were greater than zero (loneliness Q^2^ = 0.184; G2 marital intimacy Q^2^ = 0.387; intergenerational solidarity Q^2^ = 0.263; sibling relationship Q^2^ = 0.258; in-law relationship Q^2^ = 0.191; grandparent-grandchild relationship Q^2^ = 0.168; children’s support Q^2^ = 0.245), confirming the model’s predictive relevance. Taken together, these fit indices support the adequacy of the model for interpreting the direct and indirect pathways.

### Measurement model assessment

Measurement model outcomes, including reliability, convergent validity, discriminant validity, and variance explained, are presented in Table 2. Reliability analysis revealed excellent internal consistency, with Cronbach’s alpha coefficients ranging from 0.792 to 0.971. Composite reliability (CR) values ranged from 0.866 to 0.974, consistently exceeding the recommended threshold of 0.70. The rho_A coefficients showed similarly robust results (0.838–0.973), confirming the reliability of all measurement scales. Convergent validity, assessed through average variance extracted (AVE), revealed mixed results across constructs. Several constructs demonstrated adequate convergent validity with AVE values exceeding the recommended threshold of 0.50. These included G1 marital intimacy (AVE = 0.689), G2 marital intimacy (AVE = 0.629), sibling relationships (AVE = 0.571), and children’s support for parents (AVE = 0.623). However, other constructs showed AVE values below the 0.50 threshold, indicating that a substantial portion of item variance was not captured by the latent construct. Loneliness showed an AVE of 0.480, falling just below the recommended threshold. Family cohesion demonstrated an AVE of 0.468, also below the acceptable level. In-law relationships exhibited the lowest AVE at 0.397, suggesting limited convergent validity. Intergenerational solidarity showed an AVE of 0.393, similarly indicating suboptimal convergent validity. Grandparent-grandchild relationships demonstrated an AVE of 0.469, marginally below the threshold. Discriminant validity was assessed using heterotrait-monotrait (HTMT) ratios, which provide a more conservative test of discriminant validity. All HTMT values were below the conservative threshold of 0.85, ranging from 0.232 to 0.636. The lowest HTMT value was observed for in-law relationships (0.232), indicating strong discriminant validity. The highest HTMT value was for sibling relationships (0.636), still well below the threshold. This confirms that discriminant validity was established for all constructs, indicating each latent construct is empirically distinct from the others in the model. The model explained substantial variance in several endogenous constructs, as indicated by R^2^ values. G2 marital intimacy showed particularly strong explanatory power (R^2^ = 0.413, adjusted R^2^= 0.395). Intergenerational family solidarity demonstrated considerable explanatory power (R^2^ = 0.291, adjusted R^2^ = 0.278). Sibling relationships also showed strong explanatory power (R^2^ = 0.286, adjusted R^2^ = 0.273). The model accounted for moderate variance in loneliness (R^2^= 0.226). In-law relationships (R^2^ = 0.214) and grandparent-grandchild relationships (R^2^ = 0.192) demonstrated moderate explanatory power. Family cohesion showed relatively weaker explanatory power (R^2^ = 0.098). G1 marital intimacy served as an exogenous variable in the model, so it’s R^2^ was not estimated. The children’s support for parents scale, while showing slightly lower reliability than other measures (α = 0.792, CR = 0.866), still met acceptable psychometric standards and explained considerable variance (R^2^ = 0.274). These results collectively confirm adequate reliability and discriminant validity for all constructs. However, convergent validity was less consistently established across all constructs, with several AVE values falling below the recommended threshold. Given the exploratory nature of this study and the modest sample size, we retained these constructs while interpreting their findings with appropriate caution. Findings involving constructs with suboptimal AVE, particularly in-law relationships and intergenerational solidarity, should be interpreted with caution and require replication in larger samples.

**Table 2:** Outcomes of the measurement model.

Latent variable	Cronbach’s alpha	CR	rho_A	R^2^	R^2^ adjusted	AVE	HTMT
Loneliness	0.865	0.903	0.865	0.226	0.171	0.480	0.366
Marital intimacy (G1)	0.971	0.974	0.973	NE	NE	0.689	0.411
Marital intimacy (G2)	0.962	0.966	0.966	0.413	0.395	0.629	0.436
Sibling relationship	0.954	0.959	0.966	0.286	0.273	0.571	0.636
Family cohesion	0.863	0.905	0.949	0.098	0.089	0.468	0.559
In-law relationship	0.962	0.964	0.964	0.214	0.191	0.397	0.232
Intergenerational family solidarity	0.896	0.910	0.902	0.291	0.278	0.393	0.587
Grandparent-grandchild relationship	0.878	0.900	0.880	0.192	0.176	0.469	0.417
Children’s support for parents	0.792	0.866	0.838	0.274	0.267	0.623	0.490

NE: not estimated, CR: composite reliability

### Structural relationships between family dynamics and loneliness

Tables 3 and 4 and Fig 1 present the structural models and hypothesis tests examining direct and indirect pathways linking family relationship variables to loneliness (L). Using PLS-SEM with 5,000 bootstrap samples, we analyzed these relationships across two generations. The results reveal distinct patterns in how marital and intergenerational bonds relate to loneliness in later life.

**Table 3:** Direct effects of association among marital intimacy (generation 1), marital intimacy (generation 2), family cohesion, in-law relationship, intergenerational family solidarity, grandparent-grandchild relationship, children’s support for parents, and loneliness.

Hypothesis	Path	Path coefficient (95% CI)	S.D.	T-Statistics	p-value	Results
H1	MI (G1) → L	−0.194 (−0.389, 0.017)	0.105	1.836	0.067	Not-supported
H2	MI (G2) → L	−0.314 (−0.556, −0.041)	0.133	2.356	0.019	Supported
H3	FC → L	−0.093 (−0.361, 0.146)	0.125	0.751	0.453	Not-supported
H4	In-law R → L	−0.028(−0.247, 0.211)	0.117	0.232	0.816	Not-supported
H5	IFS → L	−0.095 (−0.305, 0.117)	0.110	0.904	0.366	Not-supported
H6	GGR → L	0.103 (−0.155, 0.353)	0.130	0.806	0.420	Not-supported
H7	CS → L	0.022 (−0.187, 0.232)	0.108	0.314	0.754	Not-supported

MI (G1): marital intimacy (generation 1), L: loneliness, MI (G2): marital intimacy (generation 2), FC: family cohesion, In-law R: in-law relationship, IFS: intergenerational family solidarity, GGR: grandparent-grandchild relationship, CS: children’s support for parents.

**Table 4:** Indirect effects of association among marital intimacy (generation 1), marital intimacy (generation 2), family cohesion, in-law relationship, intergenerational family solidarity, grandparent-grandchild relationship, children’s support for parents, and loneliness.

Hypothesis	Path	Path coefficient (95% CI)	S.D.	T-Statistics	p-value	Results
H1	FC → CS → L	0.011 (−0.108, 0.126)	0.059	0.301	0.764	Not-supported
H2	MI (G1) → FC → CS → L	0.003 (−0.038, 0.042)	0.020	0.276	0.782	Not-supported
H3	MI (G1) → FC → L	−0.030 (−0.068, 0.105)	0.043	0.689	0.491	Not-supported
H4	CS → GGR → L	0.025 (−0.040, 0.110)	0.037	0.631	0.528	Not-supported
H5	FC → CS → GGR → L	0.013 (−0.020, 0.059)	0.020	0.618	0.537	Not-supported
H6	MI (G1) → FC → CS → GGR → L	0.004 (−0.007, 0.019)	0.007	0.566	0.572	Not-supported
H7	CS → IFS → GGR → L	0.012 (−0.022, 0.050)	0.018	0.667	0.505	Not-supported
H8	FC → CS → IFS → GGR → L	0.006 (−0.012, 0.028)	0.010	0.640	0.552	Not-supported
H9	MI (G1) → FC → CS → IFS → GGR → L	0.002 (−0.004, 0.009)	0.003	0.602	0.547	Not-supported
H10	IFS → GGR → L	0.031 (−0.053, 0.126)	0.043	0.683	0.485	Not-supported
H11	FC → IFS → GGR → L	0.008 (−0.013, 0.041)	0.013	0.488	0.626	Not-supported
H12	MI (G1) → FC → IFS → GGR → L	0.002 (−0.004, 0.013)	0.004	0.492	0.622	Not-supported
H13	CS → In-law R → L	0.001 (−0.034, 0.041)	0.017	0.096	0.923	Not-supported
H14	FC → CS → In-law R → L	0.001 (−0.019, 0.022)	0.009	0.093	0.926	Not-supported
H15	MI (G1) → FC → CS →In-law R → L	0.000 (−0.006, 0.016)	0.003	0.086	0.931	Not-supported
H16	FC → In-law R → L	−0.001 (−0.043, 0.022)	0.011	0.021	0.983	Not-supported
H17	MI (G1) → FC → In-law R → L	−0.001 (−0.006, 0.008)	0.004	0.019	0.984	Not-supported
H18	CS → MI (G2) → In-law R → L	0.001 (−0.026, 0.023)	0.010	0.151	0.880	Not-supported
H19	FC → CS → MI (G2) → In-law R → L	0.001 (−0.010, 0.012)	0.005	0.144	0.885	Not-supported
H20	MI (G1) → FC → CS → MI (G2) → In-law R → L	0.000 (−0.004, 0.004)	0.002	0.133	0.894	Not-supported
H21	MI (G1) → MI (G2) → In-law R → L	0.006 (−0.049, 0.057)	0.027	0.221	0.825	Not-supported
H22	FC → SR → MI (G2) → In-law R → L	0.001 (−0.010, 0.015)	0.006	0.201	0.841	Not-supported
H23	MI (G1) → FC → SR → MI (G2) → In-law R → L	0.000 (−0.004, 0.005)	0.002	0.188	0.851	Not-supported
H24	MI (G2) → In-law R → L	0.015 (−0.106, 0.129)	0.061	0.217	0.828	Not-supported
H25	SR → MI (G2) → In-law R → L	−0.004 (−0.042, 0.029)	0.017	0.180	0.857	Not-supported
H26	MI (G1) → SR → MI (G2) → In-law R → L	0.001 (−0.009, 0.015)	0.005	0.137	0.891	Not-supported
H27	CS → IFS → L	−0.039 (−0.141, 0.046)	0.048	0.825	0.409	Not-supported
H28	FC → CS → IFS → L	−0.021 (−0.072, 0.030)	0.026	0.784	0.433	Not-supported
H29	MI (G1) → FC → CS → IFS → L	−0.007 (−0.029, 0.008)	0.009	0.681	0.496	Not-supported
H30	FC → IFS → L	−0.020 (−0.086, 0.033)	0.030	0.706	0.480	Not-supported
H31	MI (G1) → FC → IFS → L	−0.007 (−0.032, 0.010)	0.010	0.653	0.514	Not-supported
H32	CS → MI (G2) → L	−0.040 (−0.128, 0.016)	0.038	0.925	0.355	Not-supported
H33	FC → CS → MI (G2) → L	−0.022 (−0.073, 0.009)	0.021	0.867	0.386	Not-supported
H34	MI (G1) → FC → CS → MI (G2) → L	−0.007 (−0.026, 0.003)	0.007	0.786	0.432	Not-supported
H35	MI (G1) → MI (G2) → L	−0.140 (−0.286, −0.024)	0.067	2.128	0.033	Supported
H36	FC → SR → MI (G2) → L	−0.024 (−0.068, 0.020)	0.022	1.278	0.201	Not-supported
H37	MI (G1) → FC → SR → MI (G2) → L	−0.008 (−0.024, 0.007)	0.008	1.153	0.249	Not-supported
H38	SR → MI (G2) → L	0.066 (−0.036, 0.196)	0.060	1.186	0.236	Not-supported
H39	MI (G1) → SR → MI (G2) → L	−0.019 (−0.071, 0.006)	0.021	0.859	0.391	Not-supported

MI (G1): marital intimacy (generation 1), L: loneliness, MI (G2): marital intimacy (generation 2), FC: family cohesion, In-law R: in-law relationship, IFS: intergenerational family solidarity, GGR: grandparent-grandchild relationship, CS: children’s support for parents.

**Fig 1:**
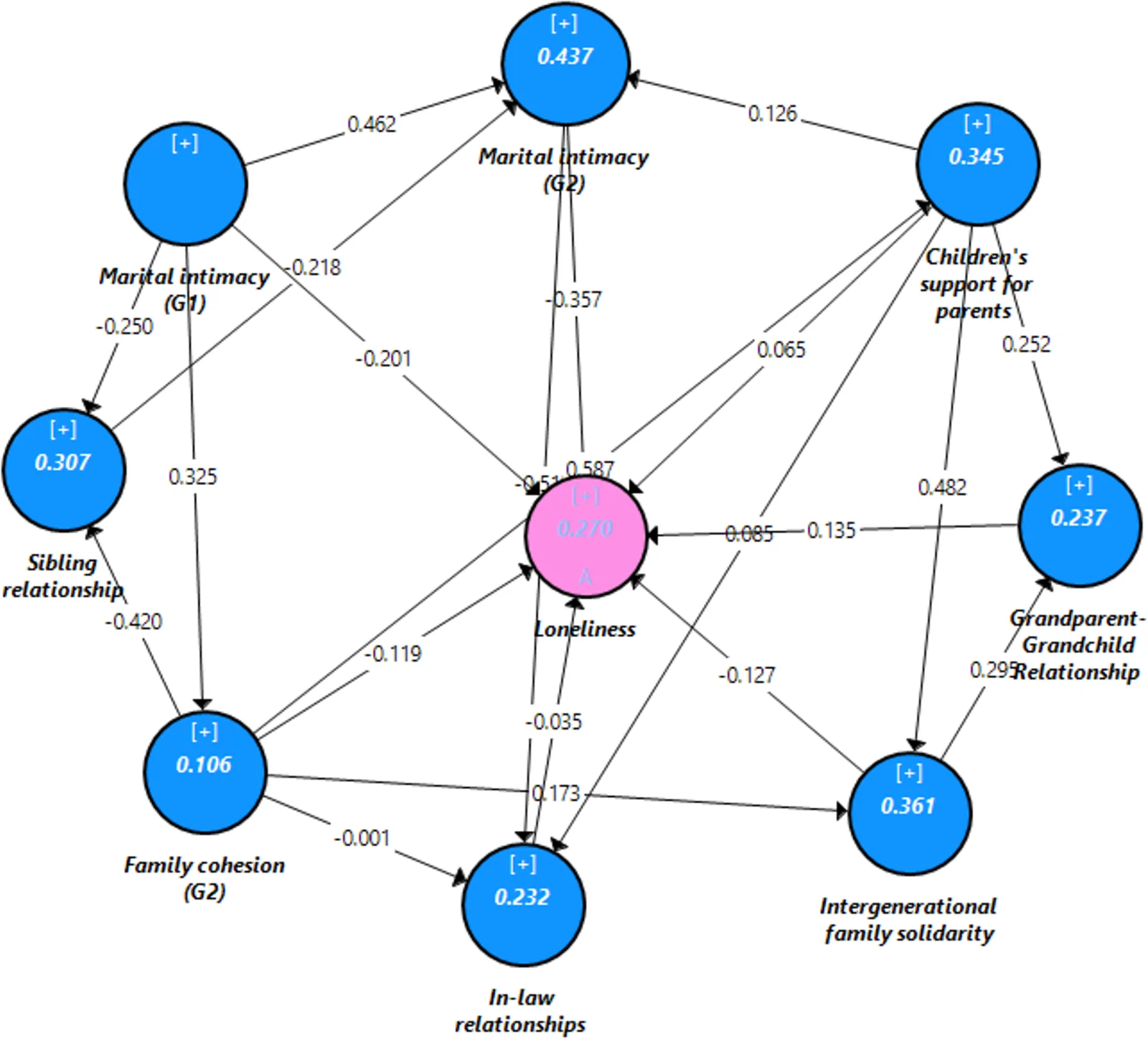
Structural models testing hypotheses on family relationships and loneliness.

### Direct effects on loneliness

Direct effects of all family relationship variables on loneliness are shown in Table 3. The analysis identified G2 marital intimacy as the only significant direct associate of loneliness (β = −0.314, p = 0.019), indicating that stronger marital bonds among adult children are associated with lower levels of loneliness in older family members. This aligns with theoretical models emphasizing the “generational pivot” role of middle-aged couples in maintaining family emotional support networks. In contrast, G1 marital intimacy showed only a marginal negative association (β = −0.194, p = 0.067), suggesting that older couples’ relationship quality may have limited direct associations with their loneliness.

Notably, none of the broader family dynamics, including family cohesion (β = −0.093, p = 0.453), in-law relationships (β = −0.028, p = 0.816), intergenerational solidarity (β = −0.095, p = 0.366), grandparent-grandchild bonds (β = 0.103, p = 0.420), or children’s support (β = 0.022, p = 0.754), demonstrated significant direct effects. These null findings challenge assumptions that generalized family connectedness is uniformly associated with lower loneliness, instead highlighting the unique importance of specific high-quality dyadic relationships.

### Indirect pathways

Indirect effects (mediation pathways) are detailed in Table 4, following the description of the model in the Results. Mediation analysis yielded one statistically significant pathway among 39 tested: G1 marital intimacy showed an indirect association with loneliness through its relationship with G2 marital intimacy (β = −0.140, p = 0.033). This supports a generational transmission model where parents’ relationship quality enhances their children’s marital bonds, which in turn is associated with lower loneliness. The comprehensive non-significance of all other indirect paths (e.g., through family cohesion, intergenerational solidarity, or in-law relationships) suggests these variables do not operate as theorized mediators. For instance, neither children’s support (β = 0.003–0.025, all p > 0.05) nor grandparent-grandchild relationships (β = 0.012–0.031, all p > 0.05) showed protective associations with loneliness, despite their hypothesized roles in family systems theory. Among the 39 indirect pathways tested, only the pathway from G1 marital intimacy through G2 marital intimacy to loneliness showed a statistically significant association (β = −0.140, p = 0.033). However, after applying Benjamini-Hochberg false discovery rate correction for multiple comparisons (q = 0.10), this pathway did not remain statistically significant (q = 0.105). Given the exploratory nature of our mediation analyses and the modest sample size, this finding should be interpreted with caution and requires replication in larger samples before firm conclusions can be drawn.

## Discussion

This study set out to explore how family communication dynamics across three generations related to loneliness in older adults, using a SEM framework. The results offer a nuanced picture, highlighting the critical role of middle-generation marital intimacy in being associated with lower elderly loneliness, while challenging commonly held assumptions about the uniform benefits of broader family ties. The strongest and only statistically significant direct effect on elderly loneliness was found in the quality of marital intimacy within the G2, that is, adult children of the elderly participants. A closer marital relationship between these middle-aged couples was associated with lower levels of loneliness among their aging parents (β = −0.314, p = 0.019). This finding supports the idea that the emotional stability and cohesion within the “pivot generation” plays a bridging role in maintaining intergenerational emotional flow, which in turn is associated with lower loneliness [27]. This pattern may partly reflect the Iranian family context, in which middle-aged couples often maintain intergenerational emotional continuity. This pattern may be less pronounced in more individualistic societies.

Interestingly, the marital quality of the older generation (G1) showed only a marginal relationship with loneliness (β = −0.194, p = 0.067). This may reflect the reduced social leverage or influence of elderly couples on the broader family network, especially when compared to their adult children who often coordinate family gatherings and maintain connections across kinship lines. Furthermore, broader relational constructs, such as family cohesion, intergenerational solidarity, in-law relationships, and even grandparent-grandchild ties, did not show significant direct associations with loneliness. While somewhat surprising, these findings suggest that general feelings of connectedness or perceived closeness may not be sufficient for lowering loneliness unless they are anchored in high-quality, stable dyadic relationships. This contrasts with our initial expectation that broader family relational factors would show stronger associations with loneliness, suggesting that emotionally meaningful dyadic bonds may be more influential than broader perceptions of family connectedness. This insight echoes a growing body of literature emphasizing the quality over quantity of social interactions in aging populations [28,29].

Prior studies have often highlighted the protective role of close family relationships in being associated with lower loneliness among older adults [30,31]. However, many of these works treat the family as a monolithic structure, without parsing out the specific dynamics that may be more or less important. Our results provide a more fine-grained analysis and indicate that not all forms of family connectedness carry the same weight. In particular, while grandparent-grandchild relationships have been celebrated in cultural and qualitative studies as sources of joy and meaning in old age [32], our quantitative model did not find them to be significant in being directly associated with lower loneliness. Similarly, the absence of significant associations from children’s support challenges assumptions embedded in traditional filial piety models, suggesting that practical or emotional support from children may not directly address the subjective experience of loneliness unless embedded within secure relational bonds.

These findings resonate with intergenerational systems theory and the concept of “emotional transmission” within family networks. The significant indirect pathway, from G1 marital intimacy to G2 intimacy, and ultimately to lower loneliness in the elderly, underscores the idea that relationship patterns are often passed down from one generation to the next [33]. This kind of generational transmission may shape how family members manage conflict, express care, and provide emotional availability. Moreover, the centrality of G2 marital intimacy supports the “generational middle” concept found in family systems literature, wherein middle-aged couples serve as emotional and logistical anchors in extended families [34]. When these anchors are stable, older family members seem to benefit indirectly by feeling more included and emotionally connected.

From a practical standpoint, these findings highlight the importance of strengthening marital and couple relationships not just for those involved but also for the well-being of older family members. Efforts to promote relational health in middle-aged adults, through counseling, communication training, or community wellness programs, may be associated with older generations. Policymakers addressing aging issues might incorporate family-based strategies into loneliness prevention. Rather than focusing solely on older adults, programs could involve adult children and offer multigenerational workshops, especially in cultures where family roles remain vital. Future research should explore why some family dynamics, like cohesion or intergenerational solidarity, may not offer the protective associations theory suggests. Qualitative studies can uncover relational subtleties, ambivalence, obligation, or emotional distance, missed in quantitative models. Longitudinal designs may clarify directionality: Does G2 marital intimacy is associated with lower loneliness, or do emotionally connected families nurture both spousal bonds and elder support? Expanding such work across cultures may reveal how norms and expectations around aging, intimacy, and intergenerational ties shape communication patterns. Comparative cross-cultural studies could enhance both theoretical understanding and practical policy. We should also be clear that our findings reflect patterns among our own participants across three generations, and we do not claim they represent entire societal generations.

### Strengths and limitations

A key strength of this study is its three-generational design, which offers a deeper view of how relational patterns evolve across family lines. Including G1, G2, and indirectly grandchildren, it provides rare insight into family communication and elderly well-being. The use of SEM enabled testing both direct and indirect pathways, revealing dynamics between family subsystems often missed in simpler models. Focusing on G2 marital intimacy helped identify a specific relationship with strong influence on elderly loneliness, suggesting a possible target for intervention. By distinguishing between spousal, intergenerational, and in-law ties, the study avoids treating family connectedness as a single concept, improving clarity and theoretical contribution.

Several limitations should be considered when interpreting the findings of this study. First, the cross-sectional design prevents any causal or directional conclusions. Although we hypothesized that G2 marital intimacy is associated with lower loneliness in older adults, the reverse direction cannot be ruled out, and most of our hypothesized pathways were non-significant, suggesting that our findings should be interpreted as exploratory and hypothesis-generating rather than confirmatory. Second, all data were collected through self-report questionnaires, which introduces potential recall bias and social desirability bias, particularly among older participants who might underreport loneliness due to stigma. Third, the sample was restricted to married older adults with at least two married children and regular family access. This criterion was necessary for the three-generation design, but it means findings do not generalize to unmarried, widowed, divorced, or childless older adults, nor to those with estranged family relationships or children living abroad. Fourth, we tested 39 indirect pathways, and after applying Benjamini-Hochberg FDR correction (q = 0.10), the single significant indirect pathway no longer reached statistical significance. This underscores the exploratory nature of our findings and the need for replication in larger samples. Fifth, all participants were from a city in northern Iran (Jouybar), a region with distinct cultural norms around family proximity and intergenerational living. Generalization to other regions or countries requires further cross-cultural validation. Sixth, the exclusion of older adults with cognitive decline, severe illness, or functional limitations, while methodologically necessary, means the findings apply only to relatively healthy, community-dwelling older adults. Finally, the study included only one adult child and one grandchild per family, which may not fully capture within-family variation in relationship quality. Telephone interviews for G2 and G3 participants may have reduced rapport compared to in-person interviews, though this is unlikely to have systematically biased the results. Given these limitations, we emphasize that our findings are preliminary and require replication in larger, more diverse populations. The exploratory nature of our results should guide future hypothesis testing rather than serve as definitive evidence.

## Conclusion

1. This study highlights the pivotal role of intergenerational marital dynamics in relating to loneliness in older adults. While traditional models stress broad family cohesion or support, our findings indicate that the quality of specific dyadic ties, especially G2 marital intimacy, has the strongest association.
2. The indirect pathway from G1 to G2 marital intimacy and, in turn, to lower elder loneliness supports a generational transmission model of relational patterns. These results enrich theory by emphasizing the emotional flow across generations and the “pivot generation’s” central role in maintaining family connection.
3. The findings may inform aging research in other societies undergoing demographic aging and changing family structures. Practically, fostering relational health in middle-aged couples may be associated with lower loneliness in aging parents. Policymakers and practitioners should consider embedding multigenerational relationship-building into public health efforts to reduce isolation among older populations.
4. Future research should examine how relational quality is transmitted over time, especially across different cultures. Ultimately, reducing loneliness among the elderly participants in our study appeared to depend not only on addressing their immediate social needs but on cultivating emotionally resilient families.
5. However, the findings should be interpreted cautiously and require replication in larger and culturally diverse populations.

## Supporting information

S1 Table Psychometric evidence for the Persian instruments used in the study. This table presents confirmatory factor analysis (CFA) results, construct validity evidence, and internal consistency estimates for all latent variables included in the measurement model.
(DOCX)

## References

[1] Salari N, Najafi H, Rasoulpoor S. The global prevalence and associated factors of loneliness in older adults: a systematic review and meta-analysis. Humanit Soc Sci Commun. 2025;12:985. doi: 10.1057/s41599-025-05304-x

[2] Gierke C, Navarro CF, Martinez M, Delgado C, Diaz-Gierke W, Diaz R, et al. The impact of social isolation and perceived loneliness in older adults: a cross-sectional descriptive Chilean study. Front Psychol. 2026;16:1683933. doi: 10.3389/fpsyg.2025.1683933 41551958 PMC12808444

[3] Puyané M, Chabrera C, Camón E, Cabrera E. Uncovering the impact of loneliness in ageing populations: a comprehensive scoping review. BMC Geriatr. 2025;25(1):244. doi: 10.1186/s12877-025-05846-4 40211165 PMC11984289

[4] Siette J, Pomare C, Dodds L, Jorgensen M, Harrigan N, Georgiou A. A comprehensive overview of social network measures for older adults: A systematic review. Arch Gerontol Geriatr. 2021;97:104525. doi: 10.1016/j.archger.2021.104525 34536656

[5] Steijvers LC, Brinkhues S, Suanet B, Stijnen MM, Hoebe CJ, Dukers-Muijrers NH. The role of social network structure and function in moderate and severe social and emotional loneliness: The Dutch SaNAE study in older adults. Heliyon. 2023;10(1):e23734. doi: 10.1016/j.heliyon.2023.e23734 38226216 PMC10788429

[6] Karmacharya I, Ghimire S, Tuladhar L, Mistry SK, Yadav OP, Prasai S, et al. Family Support Paradox: Exploring Family Support and Life Satisfaction Among Older Adults in Rural Eastern Nepal. Behav Sci (Basel). 2025;15(4):411. doi: 10.3390/bs15040411 40282033 PMC12024343

[7] Xu Q, Xiong RM, Zhao M, Wang H. The structure, function, and quality of the social convoy for improvements in depressive symptoms in urban and rural China: A 3-year longitudinal cohort study. Int Psychogeriatr. 2025;37(1):100004. doi: 10.1016/j.inpsyc.2024.100004 39924271

[8] Silverstein M, Fingerman KL, Suitor JJ. Intergenerational relationships and family support: evidence for health and well-being in studies supported by the national institute on aging. Gerontologist. 2025;65(3):gnaf003. doi: 10.1093/geront/gnaf003 39780401

[9] Liu C, Bai X. The influence of parent- and adult child-level factors on intergenerational relationship quality: A study of Chinese families with multiple children in Hong Kong. J Cross Cult Gerontol. 2023;38(1):19–37. doi: 10.1007/s10823-022-09467-x 36692664 PMC9974718

[10] Torabian M, Zanjari N, Fadayevatan R, Froughan M, Harouni GG. The intergenerational relationship patterns between aging parents and their adult children. J Family Med Prim Care. 2022;11(9):5464–72. doi: 10.4103/jfmpc.jfmpc_1972_21 36505519 PMC9730968

[11] Branje S, Geeraerts S, de Zeeuw EL, Oerlemans AM, Koopman-Verhoeff ME, Schulz S, et al. Intergenerational transmission: Theoretical and methodological issues and an introduction to four Dutch cohorts. Dev Cogn Neurosci. 2020;45:100835. doi: 10.1016/j.dcn.2020.100835 32823179 PMC7451818

[12] Mpofu E, Zhan R-F, Yin C, Brock K. Qualities of older adults’ family and friendship relationships and their association with life satisfaction. Geriatrics (Basel). 2024;9(2):49. doi: 10.3390/geriatrics9020049 38667516 PMC11050695

[13] Daniels AD, Bryan J. Resilience despite complex trauma: family environment and family cohesion as protective factors. Family J. 2021;29(3):336–45. doi: 10.1177/10664807211000719

[14] Silverstein M, Bengtson VL, Litwak E. Theoretical approaches to problems of families, aging, and social support in the context of modernization. The Need for Theory. Routledge. 2020. p. 181–98. doi: 10.4324/9781315230849-14

[15] Abramowska-Kmon A, Timoszuk S. Family network, wellbeing and loneliness among odler adults in Poland. SD. 2020;(1(177)). doi: 10.33119/sd.2020.1.1

[16] PASS 21 Power Analysis and Sample Size Software. Kaysville, UT, USA: NCSS, LLC. 2021.

[17] Hosseini L, Froelicher ES, Sharif Nia H, Ashghali Farahani M. Psychometrics of Persian version of the 11 items De Jong Gierveld loneliness scale among an Iranian older adults population. BMC Public Health. 2021;21(1):2056. doi: 10.1186/s12889-021-12068-x 34758810 PMC8578916

[18] Silverstein M, Giarrusso R. Aging and family life: a decade review. J Marriage Fam. 2010;75(5):1039–58. 10.1111/j.1741-3737.2010.00749.x PMC3427733 22930600

[19] Salehi Moghadam F, Nikorad M, Moharer Sanagouye G, Madadi P, Alimardani S. Predicting marital intimacy based on infertility stigma and coping strategies in infertile women. Appl Fam Ther J. 2024;5(1):229–37. doi: 10.61838/kman.aftj.5.1.25

[20] Momtaz YA, Vidouje MM, Foroughan M, Sahaf R, Laripour R. Grandparents - Grandchildren Relationship in Iran. Clin Pract Epidemiol Ment Health. 2018;14:296–303. doi: 10.2174/1745017901814010296 PMC6407660 30972129

[21] Samani S. Developing a family process scale for the Iranian families. IJPCP. 2008;14(2):162–8. http://ijpcp.iums.ac.ir/article-1-466-fa.html

[22] Hudson WW, MacNeil G, Dierks J. Index of Brother and Sister Relations (IBR/ISR). Walmyr Publishing Company. 1995. https://shop.walmyr.com/pdf/IBRSAMPLE.pdf

[23] Hasannia S, Farahbakhsh K, Karamrazi H, Doostian Y, Vazpour S. Sibling conflicts, gender and parenting patterns in students. Couns Cult Psychother. 2014;5(19):18–32. https://journals.atu.ac.ir/article_615.html

[24] Ghasemi S, Etemadi O, Sa A. Pathology of couples and in-law family interactions: A qualitative study. J Qual Res Health Sci. 2016;5(3):250–62. https://jqr1.kmu.ac.ir/article_90863.html

[25] Modiri F. The study of adult children’s supports for their parents and the influencing factors in Tehran. Iran Popul Stud J. 2020;6(2):3–32.

[26] Modaresifar M, Abolfathi Momtaz Y, Delbari A. Validity and reliability of the Persian version of relationship of grandchild- grandparents. Salmand: Iranian J Ageing. 2020;15(1):42–53.

[27] Andina T, Domenicucci J. States and intergenerational bonds. estetica. 2018;68:123–30. doi: 10.4000/estetica.3535

[28] Norlin J, McKee KJ, Lennartsson C, Dahlberg L. Quantity and quality of social relationships and their associations with loneliness in older adults. Aging Ment Health. 2025;29(7):1198–208. doi: 10.1080/13607863.2025.2460068 39951254

[29] Park J-H, Kang S-W. Social interaction and life satisfaction among older adults by age group. Healthcare (Basel). 2023;11(22):2951. doi: 10.3390/healthcare11222951 37998443 PMC10671417

[30] Spitzer N, Segel-Karpas D, Palgi Y. Close social relationships and loneliness: The role of subjective age. Int Psychogeriatr. 2022;34(7):651–5. doi: 10.1017/S1041610219001790 31708003

[31] Marcelino KGS, Braga L de S, Lima-Costa MF, Torres JL. Family characteristics and loneliness among older adults: evidence from the Brazilian longitudinal study of aging (ELSI-Brazil). Rev Bras Epidemiol. 2024;27:e240054. doi: 10.1590/1980-549720240054 39607137 PMC11654287

[32] Lai DWL, Li J, Bai X. To be or not to be: relationship between grandparent status and health and wellbeing. BMC Geriatr. 2021;21(1):204. doi: 10.1186/s12877-021-02052-w 33761888 PMC7989105

[33] Hou Y, Suitor JJ, Gilligan M. Intergenerational transmission of relationship quality in later-life families. J Marriage Fam. 2023;85(2):539–55. doi: 10.1111/jomf.12884 36936541 PMC10022701

[34] Zhang Y, Zang W. Do the marital statuses of adult offspring affect their parent’s mental health? Empirical evidence from China. Int J Environ Res Public Health. 2022;19(16):10133. doi: 10.3390/ijerph191610133 36011778 PMC9408382

