# Supplementary material for: The relationship between family communication networks and elderly loneliness: A structural equation modeling approach across three generations
Source: PLoS One. 2026 Oct 5;21(10):e0358707. doi: 10.1371/journal.pone.0358707 (PMC13637883; doi:10.1371/journal.pone.0358707)
Supplement: Supplementary file 1 [file pone.0358707.s001.docx]

**Supplementary Table 1.** Psychometric evidence for the Persian instruments used in the study

| **Instrument** | **Original Source** | **Persian Validation Reference** | **Translation / Adaptation Procedure** | **Factor Structure** | **Construct Validity Evidence** | **Reliability** |
| --- | --- | --- | --- | --- | --- | --- |
| **Marital Intimacy Scale (G1 & G2)** | Thompson & Walker (1983) | Salehi Moghadam et al. (2024) | Forward–backward translation; content and face validity established through expert review | Unidimensional (17 items) | CFI = 0.91, RMSEA = 0.07.  Construct validity was evidenced by significant positive correlations with marital satisfaction (r = 0.62) and negative correlations with marital conflict (r = -0.55) | Original validation: α = 0.91–0.97; test–retest r = 0.92  Present study: α = 0.96 |
| **De Jong Gierveld Loneliness Scale** | De Jong Gierveld (1985) | Hosseini et al. (2021) | Forward–backward translation following the WHO translation protocol | Two-factor structure (emotional and social loneliness; 8 items) | CFA confirmed the two-factor structure (CFI = 0.92, RMSEA = 0.06); convergent validity supported by significant correlations with depression and social support measures | Original validation: α > 0.80 Present study: α = 0.865 |
| **Intergenerational Solidarity Questionnaire** | Based on the Intergenerational Solidarity Model (Bengtson & Schrader, 1982; Silverstein & Bengtson, 1997) | Momtaz et al. (2018) | Originally developed in Persian; content validity established through expert review | Three-factor structure (emotional, associational, and functional solidarity; 16 items) | CFA supported the three-factor model (CMIN/df = 2.51, GFI = 0.924, CFI = 0.921, RMSEA = 0.063) | Original validation: α = 0.86 (subscales: 0.60–0.85)  Present study: α = 0.871 |
| **Family Cohesion Scale** | Inspired by Olson's Circumplex Model (1999).  Olson, D. H. (1999). Marriage and the Family: Diversity and Strengths (3rd ed.). Mayfield Publishing Company. | Original Iranian instrument Samani (2008) | Developed directly in Persian for the Iranian population; content validity established through expert review | Multidimensional structure comprising 8 subscales (28 items) | Convergent: r = 0.56 with Family Process Scale (p < 0.001). Discriminant: r = -0.24 with Marital Problems Scale (p < 0.05). Known-groups: divorced vs. intact families (t = 3.96, df = 58, p < 0.001). Factor analysis: 8 subscales, inter-factor r = 0.15–0.73, item-total r = 0.64–0.79. | Original validation: α = 0.79; test–retest r = 0.72–0.91 Present study: α = 0.863 |
| **Sibling Relationship Index** | Hudson, MacNeil & Dierks (1995) | CFA was performed to confirm the factor structure, which has not been previously examined in Persian | Used in Persian-speaking populations; The IBR/ISR has been used in several Iranian studies.  -https://jmums.mazums.ac. ir/article-1-10900-fa.html  -[https://www.magiran.com](https://www.magiran.com/)  /paper/1897089  -[https://ris.ui.ac.ir](https://ris.ui.ac.ir/) | Unidimensional (25 items) | Original instrument demonstrated content, construct, factorial, and known-groups validity; Present study: CFA supported the one-factor model (CMIN/df = 1.89, GFI = 0.91, CFI = 0.92, RMSEA = 0.058) | Original validation: α ≥ 0.90  Present study: α = 0.954 |
| **In-law Interaction Questionnaire** | Developed through qualitative research | Ghasemi et al. (2016) | Originally developed in Persian through qualitative research and expert review | Five-domain structure (15 items) | Content validity established during instrument development; Present study: CFA supported the five-domain structure (CMIN/df = 2.18, GFI = 0.91, CFI = 0.91, RMSEA = 0.064) | Original validation: α = 0.94–0.96; test–retest r = 0.88–0.93 Present study: α = 0.962 |
| **Children's Support for Parents Scale** | Developed based on NSFH and SHARE surveys | Modiri (2020) | Originally developed in Persian; items adapted from NSFH and SHARE; content validity established through expert review | Three-factor structure (instrumental, financial, and emotional support; 7 items) | Content validity established through expert review; EFA supported the three-factor structure; Present study: CFA confirmed the three-factor model (CMIN/df = 1.95, GFI = 0.92, CFI = 0.93, RMSEA = 0.058) | Original validation: α = 0.79–0.87 (financial support α = 0.63) Present study: α = 0.792 |
| **Grandparent–Grandchild Scale** | Elder & Conger (2000).  Elder, G. H., Jr., & Conger, R. D. (2000). Children of the Land: Adversity and Success in Rural America. University of Chicago Press | Modaresifar et al. (2020) | Forward backward translation by two bilingual translators (one with gerontology expertise), back translation by two new translators, expert committee review (four translators plus gerontology and psychology specialists), and pilot testing on 30 students; items revised for clarity and comprehensibility | Two-factor structure (emotional connectedness and participatory engagement; 10 items) | Content validity: CVI = 0.98; CFA supported the two-factor model (GFI = 0.964, AGFI = 0.938, RMSEA = 0.059, χ²/df = 2.504); all items significantly loaded on their respective factors | Original validation: α = 0.89; ; emotional dimension: α = 0.84; participation dimension: α = 0.83 Present study: α = 0.878 |
