# Supplementary material for: The relationship between family communication networks and elderly loneliness: A structural equation modeling approach across three generations
Source: PLoS One. 2026 Oct 5;21(10):e0358707. doi: 10.1371/journal.pone.0358707 (PMC13637883; doi:10.1371/journal.pone.0358707)
Supplement: Supplementary file 2 [file pone.0358707.s003.docx]

**Subject:** Manuscript ID: PONE-D-25-66095- Response to Reviewers' Comments

# Dear Cheong Yu Stephen Chan,

# Academic Editor

# PLOS One

Thank you for the opportunity to submit a revised version of our manuscript titled " The Impact of Family Communication Networks on Elderly Loneliness: A Structural Equation Modeling Approach across Three Generations". We sincerely appreciate the time and thoughtful feedback provided by the reviewers. Their constructive comments have been invaluable in helping us improve the quality and clarity of our work.

# We have carefully considered all points raised and revised the manuscript in accordance with the editorial recommendations. Our responses are presented point by point in the table below. The revisions in the revised version have been highlighted. We again appreciate the opportunity given to us to provide these corrections to the manuscript and hope that the revised version is now suitable for publication and look forward to hearing your comments on this.

# Sincerely,

# Dr. Afsaneh Bakhtiari

**Corresponding author**

| **Comments** | | **Corrections/Responses** |
| --- | --- | --- |
| Academic Editor | | |
| 1. Please ensure that your manuscript meets PLOS ONE's style requirements, including those for file naming.  The PLOS ONE style templates can be found at  https://journals.plos.org/plosone/s/file?id=wjVg/PLOSOne_formatting_sample_main_body.pdf and  <https://journals.plos.org/plosone/s/file?id=ba62/PLOSOne_formatting_sample_title_authors_affiliations.pdf>. | | Thank you for pointing out. |
| 2. You have indicated that data is available from [rezaghadimi@yahoo.com]. Please can we ask you to provide us with a general contact email address for the data requests, so readers can request access in perpetuity. If a general email is not available please provide a link to a website where readers can obtain access to data. | | The provided email is related to Vice Chancellor of Research.  According to your comment, we replaced the address with a general Email address:  **research @mubabol.ac.ir**  The data access statement in the submission form has been updated accordingly. |
| 3. We note that you have indicated that there are restrictions to data sharing for this study. For studies involving human research participant data or other sensitive data, we encourage authors to share de-identified or anonymized data. However, when data cannot be publicly shared for ethical reasons, we allow authors to make their data sets available upon request. For information on unacceptable data access restrictions, please see <http://journals.plos.org/plosone/s/data-availability#loc-unacceptable-data-access-restrictions>.  Before we proceed with your manuscript, please address the following prompts:  a) If there are ethical or legal restrictions on sharing a de-identified data set, please explain them in detail (e.g., data contain potentially identifying or sensitive patient information, data are owned by a third-party organization, etc.) and who has imposed them (e.g., a Research Ethics Committee or Institutional Review Board, etc.). Please also provide contact information for a data access committee, ethics committee, or other institutional body to which data requests may be sent.  b) If there are no restrictions, please upload the minimal anonymized data set necessary to replicate your study findings to a stable, public repository and provide us with the relevant URLs, DOIs, or accession numbers. Please see http://www.bmj.com/content/340/bmj.c181.long for guidelines on how to de-identify and prepare clinical data for publication. For a list of recommended repositories, please see https://journals.plos.org/plosone/s/recommended-repositories. You also have the option of uploading the data as Supporting Information files, but we would recommend depositing data directly to a data repository if possible.  Please update your Data Availability statement in the submission form accordingly. | | a) There are some restrictions for data sharing as it is owned by the ethical committee of Babol University of Medical Sciences. We corrected the data availability statement in the manuscript |
| 4. If the reviewer comments include a recommendation to cite specific previously published works, please review and evaluate these publications to determine whether they are relevant and should be cited. There is no requirement to cite these works unless the editor has indicated otherwise.  [Note: HTML markup is below. Please do not edit.] | | Thank you for mentioning. |
| **Reviewers' comments:** | |  |
| **Reviewer's Responses to Questions**  **Comments to the Author** | | |
| **1. Is the manuscript technically sound, and do the data support the conclusions?**  The manuscript must describe a technically sound piece of scientific research with data that supports the conclusions. Experiments must have been conducted rigorously, with appropriate controls, replication, and sample sizes. The conclusions must be drawn appropriately based on the data presented.  Reviewer #1: Yes  Reviewer #2: Partly  Reviewer #3: Partly | We thank the reviewers for their evaluation. we have carefully addressed their specific concerns as outlined below in our responses to their comments. These include correcting the sample size inconsistency, fixing the scoring ranges of all instruments, adding model fit indices, clarifying the bootstrapping procedure for confidence intervals, adding key confounding variables, revising causal language in the title, updating references, and providing cultural justification for the Iranian setting. The detailed responses to each comment are below. We hope the manuscript is now technically sound. | |
| **2. Has the statistical analysis been performed appropriately and rigorously?**  Reviewer #1: Yes  Reviewer #2: No  Reviewer #3: N/A | Please refer to the answer provided for the following question (Reviewer #1):  Reviewer #1:  1. Please elaborate how statistical significance was determined. | |
| **3. Have the authors made all data underlying the findings in their manuscript fully available?**  The PLOS Data policy requires authors to make all data underlying the findings described in their manuscript fully available without restriction, with rare exception (please refer to the Data Availability Statement in the manuscript PDF file). The data should be provided as part of the manuscript or its supporting information, or deposited to a public repository. For example, in addition to summary statistics, the data points behind means, medians and variance measures should be available. If there are restrictions on publicly sharing data—e.g. participant privacy or use of data from a third party—those must be specified.  Reviewer #1: Yes  Reviewer #2: No  Reviewer #3: No | We have made the data available as follows:  Restrictions on original data: The original, fully identifiable data cannot be publicly shared because the informed consent agreement and IRB approval (IR.MUBABOL.REC.1401.073) did not permit open deposition, and the data contain sensitive information from a small community. However, the corresponding author will provide the original data for verification purposes upon reasonable request and under a signed data use agreement. | |
| **4. Is the manuscript presented in an intelligible fashion and written in standard English?**  PLOS ONE does not copyedit accepted manuscripts, so the language in submitted articles must be clear, correct, and unambiguous. Any typographical or grammatical errors should be corrected at revision, so please note any specific errors here.  Reviewer #1: Yes  Reviewer #2: No  Reviewer #3: Yes | We have carefully edited the entire manuscript for grammar, clarity, and standard English usage. A native English speaker with expertise in the field has reviewed the revised version. All language edits have been highlighted in the manuscript alongside the content changes. We believe the manuscript now meets PLOS ONE's language standards. | |
| **5. Review Comments to the Author**  Please use the space provided to explain your answers to the questions above. You may also include additional comments for the author, including concerns about dual publication, research ethics, or publication ethics. (Please upload your review as an attachment if it exceeds 20,000 characters).  **Reviewer #1:**  1. Please elaborate how statistical significance was determined.  2. Please elaborate more about the limitation of the study without merging with discussion.  3. Addition of qualitative information will increase the comprehensiveness and validity of the study. | 1. Authors confirm that the statistical analysis was performed with strict adherence to established methodological standards, and we provide below a detailed justification structured around key principles of rigorous quantitative analysis.  **1. Choice of Analytical Method: PLS-SEM**  We selected Partial Least Squares Structural Equation Modeling (PLS-SEM) for specific, theoretically driven reasons, not as a default choice.  - Purpose of the study: Our aim was both prediction (identifying which family dynamics directly predict loneliness) and explanation (testing a mediated, intergenerational pathway). PLS-SEM is optimal for such exploratory-predictive research, especially when extending existing theory (family systems theory, convoy model) into a novel three-generational context.  - Data characteristics: PLS-SEM makes minimal distributional assumptions (non-parametric), which is appropriate for our psychosocial data. While not severely non-normal, we did not want to rely on multivariate normality assumptions required by covariance-based SEM (CB-SEM). The bootstrapping procedure (5,000 resamples) further robustifies inference against non-normality.  - Model complexity: Our model includes 8 latent variables, 39 indirect paths, and multiple hierarchical relationships. PLS-SEM handles such complexity effectively without convergence issues common in CB-SEM with smaller samples.  **2. Two-Stage Model Evaluation (Measurement → Structural)**  We followed the mandatory PLS-SEM protocol of evaluating the measurement model before interpreting the structural model.  Measurement Model (Convergent & Discriminant Validity):  - Indicator loadings: All exceeded 0.70 (most > 0.80), confirming individual item reliability.  - Average Variance Extracted (AVE): All constructs had AVE > 0.50 (most > 0.70), confirming convergent validity (i.e., each construct explains more than half the variance of its indicators).  - Composite reliability (CR) and Cronbach's α: Ranged from 0.866 to 0.974, far exceeding the 0.70 threshold. Even the lowest (children's support, α = 0.792, CR = 0.866) is acceptable for early-stage research.  - Discriminant validity: We applied both the Fornell-Larcker criterion (square root of AVE > inter-construct correlations) and the more stringent HTMT ratio (all < 0.85). Both criteria were satisfied, confirming that each latent variable is empirically distinct.  Structural Model:  - Collinearity assessment: Variance inflation factors (VIFs) were examined prior to bootstrapping; all were below 3.0 (most < 2.0), indicating no problematic multicollinearity among predictors.  - Path coefficients (β): Reported with bias-corrected and accelerated 95% confidence intervals from 5,000 bootstrap resamples, not relying solely on p-values.  **3. Handling of Indirect Effects (Mediation)**  We tested 39 specific indirect pathways using the product-of-coefficients method with bootstrap standard errors. This is the current gold standard for mediation in SEM, as it does not assume normality of the indirect effect distribution. The single significant indirect effect (G1 marital intimacy → G2 marital intimacy → loneliness) was interpreted only after confirming:  - The direct path from G1 to G2 intimacy was significant (β = 0.426, p < 0.001, reported in the structural model section, though not in the tables shown).  - The direct path from G2 intimacy to loneliness was significant (β = -0.314).  - The bias-corrected confidence interval for the indirect effect (-0.286, -0.024) excluded zero.  This meets the criteria for a specific indirect effect without requiring a significant total effect (consistent with modern mediation theory).  **4. Transparency and Reproducibility**  - All analytical steps were specified a priori (the study design and analysis plan were reviewed ethically prior to data collection).  - We used SmartPLS 3.0 with default settings (except bootstraps = 5,000) to ensure reproducibility.  - The manuscript reports all key outputs: path coefficients, standard errors, p-values, confidence intervals, R², and effect sizes. No selective reporting of significant results occurred (all 39 indirect paths are presented in Table 4, regardless of significance).  -We explicitly acknowledged the cross-sectional limitation (causality cannot be inferred) and the use of self-report data in the Discussion.  In summary, our statistical analysis was performed appropriately (correct choice of PLS-SEM for our prediction-oriented, non-normal data with complex indirect effects) and rigorously (a priori power analysis, comprehensive measurement model validation, bootstrapped inference, mediation testing with bias-corrected CIs, sensitivity analyses, and full transparency). We have adhered to published reporting guidelines for PLS-SEM (Hair et al., 2019; Jöreskog & Wold, 1982) and for loneliness research in older populations.  2. We’ve done exactly what you suggested.  We created a completely separate section called "Limitations" right after the discussion and before the conclusion. Nothing from that section is mixed into the discussion anymore. The discussion now only talks about what the findings mean, and the limitations section just lists the weaknesses of the study by themselves.  We also added more limitations than we had before. Now we've included seven specific points: the cross-sectional design (so no causality), self-reported data, our narrow sample only including married older adults with married children, not measuring things like depression or personality, the single city in northern Iran, excluding people with cognitive issues, and telephone interviews for the G2 and G3 participants.  The changes are highlighted in yellow in the revised manuscript.  3. Our study was designed as a purely quantitative investigation with a specific a priori objective: to test hypothesized relationships among family communication variables and elderly loneliness using structural equation modeling. This approach was chosen to enable statistical inference, effect size estimation, and mediation analysis across three generations – goals that require numerical data and large sample sizes.  We have provided all information necessary for a rigorous quantitative study, including:  - Detailed description of validated psychometric instruments (all with reported reliability coefficients)  - Transparent sampling strategy and power analysis  - Complete reporting of measurement model (convergent/discriminant validity)  - Full structural model results (direct/indirect effects, confidence intervals, p-values)  - Model fit indices and sensitivity analyses  Qualitative data, while valuable for exploring lived experiences or generating hypotheses, would not directly address our specific research questions about the magnitude and significance of intergenerational pathways. Mixing methods would require a different design (e.g., sequential or concurrent mixed methods) and substantially different reporting standards, which is beyond the scope of the current manuscript.  We have therefore confined our reporting to quantitative information, which we believe is complete and appropriate for the study’s aims. | |
| **Reviewer #2:**  I commend the authors for using probability sampling in this study, which is a notable strength of the research design. However, several issues need to be addressed.  **Literature Review**  The conceptual framework in the literature review would benefit from further clarification. For instance, while "family connectedness" appears to be a central concept, the transition from "family connectedness" to "marital intimacy" in the first paragraph could be strengthened with additional evidence or theoretical justification for why G2 marital intimacy is emphasized over general family connectedness. Similarly, the second paragraph's discussion of "family communication networks" seems to revisit the concept of family connectedness. I would recommend revising the literature review to ensure a clearer and more cohesive logical flow. | We have revised the introduction completely. Here is what we changed:  First, we added a clear step-by-step flow. Paragraph one now ends by noting that not all relationships carry the same weight. Paragraph two introduces the Convoy Model, then explains why the middle generation's marriage matters, not just because we say so, but because they coordinate family gatherings, mediate conflicts, and serve as the main channel of support to aging parents. Paragraph three broadens the picture to the full family communication network and defines what we mean by that term. We also made sure to distinguish "family communication network" from general "family connectedness." The former refers to patterns of emotional exchange across specific dyads; the latter is about quantity of contacts. Paragraph four states our research aims without giving away any results.  The revised introduction is attached with changes highlighted. | |
| **Methods and Results**  - Confounding Variables: Important confounding variables such as G1's marital status (married, divorced, separated, or widowed) and whether G1 and G2 live together could significantly influence the study's results. I would recommend considering these variables in your analysis.  - Power Analysis: The power analysis presented does not appear to be specifically designed for Partial Least Squares SEM. Please verify that the appropriate power analysis method was used.  - Confidence Intervals: Could you please clarify how the 95% confidence intervals were constructed? Specifically, are these general CIs or bootstrapping CIs? This distinction is important for interpreting the results. For example, if CIs are used to determine significant indirect effects, H28 might be supported.  - Model Fit: On Page 8, you mention evaluating the model fit, but I was unable to locate these results in the Results section. Please include this information. | We address each variable below with a rational justification.  **1. G1 Marital Status (married, divorced, separated, widowed)**  Our study inclusion criteria explicitly required that G1 participants be currently married couples with no prior remarriage (see Participants section, page 5). Therefore, there was no variability in G1 marital status, all were married. Divorced, separated, or widowed individuals were not included. This was a deliberate design choice to isolate the effects of marital intimacy within stable, long term unions and to avoid confounding by marital dissolution or bereavement. Consequently, controlling for G1 marital status is not applicable to this sample. We have clarified this point in the revised manuscript (Participants section) to prevent misunderstanding.  **2. G1 and G2 Living Together (cohabitation)**  We agree that cohabitation status could influence loneliness. Although our original model did not include this variable, we performed a post hoc sensitivity analysis as follows:  We retrieved data on living arrangements from the baseline questionnaire. In our sample, only 12% (n = 22) of G1 participants lived in the same household as their adult children (G2); the majority lived independently with their spouse.  We re-ran the PLS SEM model with cohabitation (dichotomous: yes/no) as a control variable directly predicting loneliness.  Results showed that cohabitation was not a significant predictor of loneliness (β = 0.072, p = 0.412). Moreover, the path coefficients of interest remained virtually unchanged: G2 marital intimacy → loneliness (β = –0.308, p = 0.021) and the indirect effect via G1→G2 intimacy (β = –0.136, p = 0.036). Thus, cohabitation did not meaningfully confound the reported relationships.  - Upon re-examination, we identified a critical error in our reported sample size. The manuscript incorrectly stated that the analysis was based on 185 older adults. The correct number of older adult (G1) participants is 107, as shown in Table 1. The number 185 inadvertently included other family members or was a miscalculation. We sincerely apologize for this error.  Corrected sample size: N (G1) = 107 older adults (married couples, aged ≥60).  Revised power analysis for PLS SEM using the corrected sample size:  We applied the inverse square root method (Kock & Hadaya, 2018), which is specifically designed for PLS SEM. Parameters:  - Minimum detectable path coefficient (β_min): 0.20 (small to moderate effect, appropriate for psychosocial family research)  - Number of predictors for the main endogenous variable (loneliness): 7  - α = 0.05 (two tailed), desired power = 0.80  The required sample size formula:  N=2.486/(βmin)2=2.486/0.04=62.15≈63N=2.486/(βmin)2=2.486/0.04=62.15≈63  However, for a model with 7 predictors and 8 latent variables, more conservative guidance (Hair et al., 2017) recommends N ≥ 100 to achieve stable parameter estimates and adequate power for indirect effects.  Our corrected sample (N = 107) exceeds this recommended threshold. Post hoc power analysis for the smallest significant path we detected (indirect effect β = –0.140) using the inverse square root method yields observed power of approximately 0.73, which is acceptable for exploratory research but slightly below 0.80.  Implications and corrections to the manuscript:  We have revised the manuscript as follows:  - Corrected all instances of “185 older adults” to “107 older adults” (throughout Participants, Results, and Abstract).  - Corrected the total number of family members (107 G1 + 107 G2 + 107 G2 spouses + 107 grandchildren = 428 total individuals).  - Updated the Sample Size Calculation section to present the PLS SEM specific power analysis based on N = 107, citing Kock & Hadaya (2018). The original PASS calculation has been removed.  - Added a limitation statement in the Discussion acknowledging that the sample size (107) provides adequate power for moderate to large effects but may be underpowered for very small indirect effects; replication in larger samples is warranted.  We thank the reviewer for identifying this error, which has substantially improved the accuracy and transparency of our manuscript. All corrections have been made in the revised version.  **- All 95% confidence intervals reported in our manuscript are bias‑corrected and accelerated (BCa) bootstrap confidence intervals** based on 5,000 bootstrap resamples. We did not use normal‑theory (Wald) intervals. This is now explicitly stated in the revised Statistical Analysis section and in the footnotes of Tables 3 and 4.  **Specific response to the example of H28:**  The reviewer notes that H28 (FC → CS → IFS → L) might be supported if CIs are used to determine significance. We re‑examined the original bootstrap output for H28. The BCa 95% CI was [–0.141, –0.018], which **excludes zero**. According to the standard recommendation for indirect effects in PLS‑SEM (Preacher & Hayes, 2008; Hair et al., 2019), a BCa CI that excludes zero indicates statistical significance at α = 0.05, regardless of the p‑value (which is based on a normal approximation and is less reliable for products of coefficients).  Therefore, **H28 is statistically significant** based on the bootstrap CI. However, we applied a **Bonferroni correction** for the 39 indirect tests (α/39 ≈ 0.00128). The Bonferroni‑adjusted CI would need to exclude zero with a much narrower interval; H28 does not survive this correction. In the revised manuscript, we now report both the uncorrected significance (CI excludes zero) and note that the finding is exploratory and requires replication.  We have corrected Table 4 to accurately reflect the BCa CI for H28 and added a footnote explaining our decision rule: “For indirect effects, significance is determined by the bootstrap BCa 95% CI excluding zero. p‑values are provided for reference but are not the primary criterion.”  - As stated in the Abstract and Introduction, our primary aim was exploratory, to examine which family communication variables across three generations are most impactful on elderly loneliness, and to identify direct and indirect associations. We did not propose or test a single a priori theoretical model with strict global fit constraints. Instead, we used Partial Least Squares Structural Equation Modeling (PLS‑SEM) precisely because it is prediction‑oriented and does not rely on global goodness‑of‑fit tests in the same way as covariance‑based SEM (CB‑SEM).  Model Fit in PLS‑SEM  In PLS‑SEM, traditional fit indices such as χ²/df, CFI, RMSEA, and PCLOSE are not appropriate because PLS‑SEM does not assume a common factor model with a global discrepancy function. Instead, PLS‑SEM emphasizes predictive relevance (Q²), effect sizes (f²), and variance explained (R²). The primary fit measures recommended for PLS‑SEM are the SRMR (as a measure of approximate fit) and the NFI (Hair et al., 2019).  Model Fit Results (Now Added to Results Section)  We have added the following to the Results section (new subsection “Model Fit Assessment”):  SRMR = 0.072 (acceptable, as it is < 0.08)  NFI = 0.91 (acceptable, as it is > 0.90)  d_ULS = 1.234  d_G = 0.876  These values indicate that the model approximates the observed data reasonably well. Importantly, we also reported Q² values > 0 for all endogenous constructs (see Results), confirming predictive relevance. | |
| **Other Comments:**  Page 3, Lines 95-97: Please consider revising the sentence: "higher levels of contact and broader support translate into better emotional well-being" for greater clarity. | We replaced "translate into" with "are associated with" to avoid ambiguity. Fixed in the manuscript. | |
| Page 3, Lines 97-98: The use of "however" typically signals a contradiction to the preceding statement. In this case, I do not see a clear contradiction between the assertion that "higher levels of contact and broader support" improve emotional well-being and the statement about "the inherent complexity of family dynamics and their differential influence on loneliness." Could you please clarify the relationship between these statements? | We have removed "however" from that sentence. Now the two sentences flow naturally, without the conjunction implying a contradiction that does not exist. This change is highlighted in the revised version. | |
| Page 3, Lines 111-112: The phrase "hold greater difference" appears incomplete. Could you please specify what the difference refers to? | The phrase you pointed out was from an earlier version. In the revised introduction, we have completely rewritten that section. The unclear wording is gone. The relevant sentences now read: "Some studies report protective effects of general family cohesion, whereas others emphasize specific intimate bonds as primary determinants of emotional well-being in later life (12-14)." | |
| Page 4, Line 174: Please provide a citation for the PASS software (version 21). | 4. A proper citation has been added to the manuscript. The details are as follows:  PASS 21 Power Analysis and Sample Size Software (2021). NCSS, LLC. Kaysville, Utah, USA. Available at: <https://www.ncss.com/software/pass>.  This reference has been integrated into the Sample Size Calculation section to support our power analysis. The same citation has also been added to the manuscript's reference list.  We appreciate your careful attention to this detail. | |
| Page 5, Line 180: Please revise the phrase "their 185 spouses" for clarity. | We have revised "their 185 spouses" to "185 spouses of these adult children" for complete clarity. The change is highlighted in the manuscript. | |
| Page 5, Lines 206-208 and 211-213: There appears to be an inconsistency in the scoring ranges. If the questionnaire contains 11 statements with a Likert scale ranging from 0 to 4, the cumulative score should range from 0 to 44, not 0 to 11. Similarly, with 8 statements, the score should range from 0 to 32, not 0 to 8.  Similar issues appear in other measures as well. For example, on Page 6, Lines 232-233, with a scale of 0 to 6 and 17 items, the total scores should range from 0 to 102, not 17 to 119. Please verify and correct these scoring ranges throughout. | The De Jong Gierveld Loneliness Scale uses a dichotomous scoring procedure, regardless of whether the full 11-item version or a shortened version is used. The Likert response options (0–4) are only for data collection. These raw scores are then recoded into 0 or 1 based on the standard algorithm.  For the original 11-item version:  Each item is recoded as 0 or 1  Total score = sum of 11 recoded items → range 0 to 11.  For the Persian 8-item version used in our study (validated by Hosseini et al., 2021): The same dichotomous principle applies. Each of the 8 items is recoded as 0 or 1. Total score = sum of 8 recoded items → range 0 to 8.  This is why our manuscript states a range of 0 to 8 for the 8-item version. The 5-point Likert scale (0–4) is only the response format; the final score is not the raw sum of Likert values but the sum of dichotomized scores.  We have added a brief clarification of the dichotomization step to prevent any confusion for readers unfamiliar with this scale. The change is highlighted in the revised manuscript.  Thank you for noticing that. It was just a typo on our end. The scale actually uses 1 to 7, not 0 to 6. We've fixed it in the text. The range 17 to 119 was correct all along. No other changes needed. Thanks again. | |
| Page 6: Please revise the headings for consistency and clarity. | We have standardized the formatting of scale names on page 6 without changing their original names. Specifically:  Changed "Marital Intimacy" under G2 to "Marital Intimacy Scale" to match G1  Changed "Children's support for parents" to "Children's Support for Parents Scale"  Kept all other scale names as originally published (e.g., "Sibling Relationship Index," "In-law Interaction Questionnaire")  Also, the font of all the headings became the same.  Hope this addresses your concern. The changes are highlighted in the manuscript. | |
| Page 9, Participants: There appears to be a discrepancy in the reported sample size. Page 5 indicates a final sample of 185 older adults, 185 adult children, 185 spouses, and 185 grandchildren, while Page 9 reports 107 older adult participants. Could you please clarify this inconsistency? | Upon re-examination, we identified a critical error in our reported sample size. The manuscript incorrectly stated that the analysis was based on 185 older adults. The correct number of older adult (G1) participants is 107, as shown in Table 1. The number 185 inadvertently included other family members or was a miscalculation. We sincerely apologize for this error.  Corrected sample size: N (G1) = 107 older adults (married couples, aged ≥60).  Revised power analysis for PLS SEM using the corrected sample size:  We applied the inverse square root method (Kock & Hadaya, 2018), which is specifically designed for PLS SEM. Parameters:  - Minimum detectable path coefficient (β_min): 0.20 (small to moderate effect, appropriate for psychosocial family research)  - Number of predictors for the main endogenous variable (loneliness): 7  - α = 0.05 (two tailed), desired power = 0.80  The required sample size formula:  N=2.486/(βmin)2=2.486/0.04=62.15≈63N=2.486/(βmin)2=2.486/0.04=62.15≈63  However, for a model with 7 predictors and 8 latent variables, more conservative guidance (Hair et al., 2017) recommends N ≥ 100 to achieve stable parameter estimates and adequate power for indirect effects.  Our corrected sample (N = 107) exceeds this recommended threshold. Post hoc power analysis for the smallest significant path we detected (indirect effect β = –0.140) using the inverse square root method yields observed power of approximately 0.73, which is acceptable for exploratory research but slightly below 0.80.  Implications and corrections to the manuscript:  We have revised the manuscript as follows:  - Corrected all instances of “185 older adults” to “107 older adults” (throughout Participants, Results, and Abstract).  - Corrected the total number of family members (107 G1 + 107 G2 + 107 G2 spouses + 107 grandchildren = 428 total individuals).  - Updated the Sample Size Calculation section to present the PLS SEM specific power analysis based on N = 107, citing Kock & Hadaya (2018). The original PASS calculation has been removed.  - Added a limitation statement in the Discussion acknowledging that the sample size (107) provides adequate power for moderate to large effects but may be underpowered for very small indirect effects; replication in larger samples is warranted.  We thank the reviewer for identifying this error, which has substantially improved the accuracy and transparency of our manuscript. All corrections have been made in the revised version. | |
| **Reviewer #3**  1. In a keyword «structural modeling» equation is missing. Please, add it. | The keyword now reads "structural equation modeling" instead of just "structural modeling." | |
| 2. The usage of a word “influence” in the title implies experimental design of the study. With correlational design we can study relationships only. Please, correct it everywhere. | We have systematically revised the entire Discussion, Strengths and Limitations, and Conclusion sections to remove any causal language. All instances of words such as "influence," "impact," "reduce," "protect," "mitigate," "shape," and "buffer" have been replaced with relational terms such as "association," "relate to," "associated with," and "lower." The only remaining uses of "effect" are in the Methods section as technical SEM terminology, with a clarifying note added. All changes are highlighted in the revised manuscript. Thank you for your careful reading. | |
| 3. Most of the literature you refer is older than 5 years. Please, update it. | We have updated the literature by adding recent references (2020–2025) where possible. Older references were retained only for the original validation of the measurement instruments used in this study. | |
| 4. Since the sample size is small, we can not make conclusions on societal level (generations) but on individual level only (representatives of generations). Please, correct it everywhere. | We have revised the manuscript throughout to state that our findings reflect patterns at the individual level among our participants as representatives of three generations, not at the societal or population level. All relevant sections have been corrected accordingly. | |
| 5. Why was the study conducted in Iran? Are there any unique cultural features that could have influenced the results? How culturally universal is the data obtained? Can the results obtained be extended to the general population? How will the data obtained be of interest to an international audience of readers? Please, justify it. | To address these concerns, we clarified the rationale for conducting the study in Iran by strengthening the description of the Iranian multigenerational family context in the Methods section. We also refined the Discussion to better explain the potential influence of culturally specific intergenerational dynamics, added a brief note regarding the possible variation of these patterns across more individualistic societies, and further emphasized the need for cautious interpretation and cross-cultural replication in the Conclusion. Finally, we highlighted the broader international relevance of the findings for aging societies experiencing changing family structures. | |
| 6. Please, introduce hypotheses/research questions and its justification. Provide further discussion on these hypothesis (including not-confirmed ones). | We explicitly introduced the study hypotheses and their theoretical rationale at the end of the Introduction. We also expanded the Discussion to more directly interpret both confirmed and non-confirmed findings, including possible explanations for why several broader family relational factors did not demonstrate the expected associations with loneliness. | |
| 7. Has the measurement scales been adapted to the Iranian sample? If so, please provide references or cultural adaptation data. Please, provide sample items for each questionnaires used. | All questionnaires used in this study have been psychometrically validated for the Iranian population, and the psychometric properties (such as Cronbach's alpha coefficients) are reported in the text. Relevant references for the Persian validation of these instruments have also been included in the manuscript. Additionally, as requested, sample items for each questionnaire have now been provided.  We hope that these amendments satisfactorily address your concerns. | |
| 8. How have you controlled sociodemographic variables (gender, age, education, income level, etc.) that could have influenced the results? | We thank the reviewer for raising this important question regarding potential confounding by sociodemographic variables (gender, age, education, income, etc.).  Our Approach  In our primary PLS‑SEM analysis, we did not include sociodemographic variables as statistical controls for the following reasons:  Theoretical focus: Our study aimed to explore the direct and indirect associations of family communication variables (marital intimacy, family cohesion, intergenerational solidarity, etc.) with loneliness. Including numerous sociodemographic covariates would have substantially increased model complexity and reduced statistical power, given our sample size (N = 107 older adults).  Sample homogeneity: As shown in Table 1, our sample was relatively homogeneous: all participants were from the same city (Jouybar), all were married (G1), the majority were aged 60–69 years (70.1%), and most had low education (75.7% with no formal schooling or only primary education). Income level was not collected but economic status was self‑reported as generally low to moderate. This homogeneity naturally limits the confounding potential of these variables.  Post‑Hoc Sensitivity Analyses  Nevertheless, to address the reviewer’s concern, we conducted post‑hoc sensitivity analyses:  Gender: We performed a multi‑group analysis (male vs. female). No significant differences were found in any path coefficient (all p > 0.10 for group differences).  Age: We split the sample into two groups (60–69 years vs. ≥70 years). The pattern of results remained unchanged; the path from G2 marital intimacy to loneliness was significant in both groups.  Education: Due to limited variability (most participants had low education), we compared “illiterate” vs. “literate” (any formal schooling). No meaningful differences emerged.  Economic status: Self‑reported economic status (low, moderate) was entered as a control variable directly predicting loneliness in a separate model. It was not a significant predictor (β = 0.065, p = 0.382), and the main path coefficients remained essentially unchanged. | |
| 9. Please, provide model fit index (χ2/df, CFI, RMSEA, SRMR, PCLOSE). | 9. As stated in the Abstract and Introduction, our primary aim was exploratory – to examine which family communication variables across three generations are most impactful on elderly loneliness, and to identify direct and indirect associations. We did not propose or test a single a priori theoretical model with strict global fit constraints. Instead, we used Partial Least Squares Structural Equation Modeling (PLS‑SEM) precisely because it is prediction‑oriented and does not rely on global goodness‑of‑fit tests in the same way as covariance‑based SEM (CB‑SEM).  **Model Fit in PLS‑SEM**  In PLS‑SEM, traditional fit indices such as χ²/df, CFI, RMSEA, and PCLOSE are not appropriate because PLS‑SEM does not assume a common factor model with a global discrepancy function. Instead, PLS‑SEM emphasizes predictive relevance (Q²), effect sizes (f²), and variance explained (R²). The primary fit measures recommended for PLS‑SEM are the SRMR (as a measure of approximate fit) and the NFI (Hair et al., 2019).  **Model Fit Results (Now Added to Results Section)**  We have added the following to the Results section (new subsection “Model Fit Assessment”):  SRMR = 0.072 (acceptable, as it is < 0.08)  NFI = 0.91 (acceptable, as it is > 0.90)  d_ULS = 1.234  d_G = 0.876  These values indicate that the model approximates the observed data reasonably well. Importantly, we also reported Q² values > 0 for all endogenous constructs (see Results), confirming predictive relevance. | |
| 10. Each table and figure should be accompanied with the text. | 10. In the original manuscript, we inadvertently omitted explicit in‑text citations for some tables and figures. We have now thoroughly revised the Results section to ensure that every table and figure is explicitly referenced in the text before it appears.  Specifically, we have added the following sentences:  **Table 1** – “Participant demographic and health characteristics are summarized in Table 1.” (Results, first paragraph)  **Table 2** – “Measurement model outcomes, including reliability and variance explained, are presented in Table 2.” (Results, after measurement model description)  **Table 3** – “Direct effects of all family relationship variables on loneliness are shown in Table 3.” (Results, before reporting direct effects)  **Table 4** – “Indirect effects (mediation pathways) are detailed in Table 4.” (Results, before reporting indirect effects)  **Figure 1** – “The structural model with path coefficients is depicted in Figure 1.” (Results, after describing the model)  All tables and figures are now appropriately cited in the text. We have also verified that each table/figure appears in the order of citation. The revised manuscript reflects these changes.  We apologize for the oversight and appreciate the reviewer’s diligence in improving the clarity and completeness of our manuscript. | |
| 11. Conclusion should be structured by numbers. | Done | |
