# Supplementary material for: The relationship between family communication networks and elderly loneliness: A structural equation modeling approach across three generations
Source: PLoS One. 2026 Oct 5;21(10):e0358707. doi: 10.1371/journal.pone.0358707 (PMC13637883; doi:10.1371/journal.pone.0358707)
Supplement: Supplementary file 3 [file pone.0358707.s004.docx]

**Dear Dr. Chan,**

**Academic Editor**

**PLOS One**

Manuscript ID: PONE-D-25-66095R1

Title: The Relationship between Family Communication Networks and Elderly Loneliness: A Structural Equation Modeling Approach across Three Generations

Thank you for your decision letter and for the opportunity to submit a revised version of our manuscript. We also appreciate the time and careful attention that both reviewers have devoted to our work. We are pleased that Reviewer #1 and Reviewer #3 confirmed that our previous revisions addressed their initial comments satisfactorily.

In this round, we have focused on the additional methodological considerations raised by Reviewer #3. While we believe our original approach was sound, we recognize that the reviewer's suggestions have helped us present our findings with greater transparency and rigor. We have therefore conducted supplementary analyses, rechecked our statistical reporting, and refined our language throughout the manuscript to ensure clarity and precision.

All changes are clearly marked using tracked changes in the revised file, and we have also provided a clean, untracked version for your convenience. We are confident that the current version represents a stronger and more complete presentation of our research.

Below, we respond to each point raised by Reviewer #3 in the order they were presented.

Thank you again for your time and consideration.

Yours sincerely,

Dr. Afsaneh Bakhtiari

Corresponding Author

| Reviewer #3 | Corrections/Responses |
| --- | --- |
| 1. Alphas and test-retest reliability are not enough for validity. This shows the internal consistency and stability, but not the validity of the Persian version of scales used. Please, provide an accurate reference to the adaptation and briefly indicate the translation procedure, factor structure, and data on constructive validity. It can be included to appendix. Judging by the description, some of the sources cited are studies using scales, rather than validating them. | The Measurements section has been revised to include the appropriate Persian validation references together with a brief summary of the translation/adaptation procedure, factor structure, construct validity, and reliability for each instrument. To keep the main text concise, comprehensive psychometric information for all measures has been provided in Supplementary Table 1, and the corresponding references have been updated accordingly. |
| 2. Comparisons of age, gender, and educational groups are not a substitute for including covariates in the model. It is better to peform sensitive analysis with age, gender, education/economic status, health, and cohabitation, and show whether the main pathway and the indirect effect change. | Sensitivity Analysis Results: We conducted a sensitivity analysis by sequentially including age, gender, education, economic status, chronic disease status, and cohabitation status as covariates in the structural model. The primary pathway (G2 marital intimacy → loneliness) and the significant indirect effect (G1 marital intimacy → G2 marital intimacy → loneliness) remained essentially unchanged: Parameter \| Original Model \| Model with Covariates \| Change; G2 MI → Loneliness (β) \| –0.314 (p = 0.019) \| –0.302 (p = 0.024) \| Minimal; G1 MI → G2 MI → Loneliness (β) \| –0.140 (p = 0.033) \| –0.135 (p = 0.039) \| Minimal None of the covariates showed significant direct associations with loneliness in the full model (all p > 0.10). Model fit indices remained comparable (SRMR: 0.072 vs. 0.075), and the magnitude of the main coefficients changed by less than 0.02. These findings confirm that the observed relationships are not confounded by sociodemographic or health-related factors. |
| 3. There are still contradictions in the text regarding the calculation of statistical power and sample size. | We have revised this section to eliminate any ambiguity and ensure consistency throughout the manuscript. Below, we provide a detailed explanation of our sample size calculation and the final number of participants included. The required sample size was determined based on the study's primary objective of examining the association between family communication networks and loneliness. A minimum correlation coefficient of 0.30 was used as the reference effect size, which was derived from prior studies on loneliness and family networks in similar populations (Abramowska-Kmon & Timoszuk, 2020) [15]. This effect size was selected as it represents a moderate association that is both clinically meaningful and theoretically plausible given the complex nature of family relationship dynamics.  Using PASS software (version 21) [16], with a two-tailed test, 95% confidence level (α = 0.05), and 80% power (β = 0.20), the initial calculation yielded a requirement of 85 elderly couples (170 individuals). This calculation was based on detecting a correlation coefficient of r = 0.30 between family communication network variables and loneliness scores.  To account for a potential 10% non-response rate or incomplete data, the target sample size was adjusted upward to 94 elderly couples (188 individuals). This conservative adjustment ensured that even with some data loss, we would maintain adequate statistical power for our primary analyses.  In practice, we successfully enrolled 107 eligible elderly couples who met all inclusion criteria. Following data collection, five cases were excluded due to incomplete questionnaires, leaving a final analytic sample of 107 older adults (representing 107 couples, as each older adult was part of a couple). It is important to clarify that the final sample of 107 older adults corresponds to 107 couples, not 107 individuals in the first generation. This number (107) exceeds our target of 94 couples, providing a margin above the minimum required sample size.  Alongside the first-generation participants (n = 107 older adults), we enrolled 107 adult children (one per family), their 107 spouses, and 107 grandchildren, resulting in a total of 428 individuals representing three generational levels within extended families. The discrepancy between the initial calculation (85 couples) and the final sample (107 couples) reflects our conservative approach to sample size planning and the successful recruitment of eligible participants who met the study's stringent inclusion criteria. |
| 4. For one indirect effect, the confidence interval excludes zero, but (p =.433), this means an error. Correction for multiple comparisons is applied inconsistently. The claimed indicators of the measuring model (loads, AVE and HTMT) are not actually presented. | We have carefully re-examined all our statistical outputs and identified the following concerns: (1) a typographical error in reporting the p-value for the significant indirect effect, (2) inconsistent application of multiple comparisons correction, and (3) insufficient presentation of measurement model indicators. We have addressed each issue comprehensively below.  Issue 1: P-value Inconsistency for the Indirect Effect  Upon careful re-examination of our SmartPLS output, we have identified that Hypothesis 28 (H28) in Table 4 contained a reporting error. We have corrected it below. Upon re-examining our original SmartPLS output, we identified the following error in Table 4: H28: FC → CS → IFS → L (Family Cohesion → Children's Support → Intergenerational Family Solidarity → Loneliness). The confidence interval reported in the original manuscript was a data entry error. The correct 95% confidence interval for H28 from our SmartPLS output is (-0.072, 0.030) which includes zero and is consistent with p = 0.433 (non-significant).  Issue 2: Correction for Multiple Comparisons  We acknowledge that testing 39 indirect pathways raises concerns about Type I error inflation. We have now applied the Benjamini-Hochberg false discovery rate (FDR) procedure to all indirect pathways. With FDR adjustment (q = 0.10), the indirect pathway through G2 marital intimacy (p = 0.033, adjusted q = 0.105) no longer reaches statistical significance at the 0.10 level. However, this result should be interpreted with consideration of two factors:  Statistical power: With a modest sample size (n = 107), the study may have been underpowered to detect smaller indirect effects, particularly after correction for multiple comparisons. The fact that this pathway showed the strongest evidence among all 39 tested pathways suggests a consistent pattern, even if it does not survive conservative correction.  Hypothesis-driven testing: The primary hypothesis of our study focused specifically on the intergenerational transmission pathway (G1 MI → G2 MI → L), which we specified a priori based on theoretical frameworks. FDR correction is conservative when applied uniformly to a large number of tests, many of which were exploratory or theoretically less important.  Issue 3: Measurement Model Indicators  We now provide a more comprehensive assessment of the measurement model. In our original manuscript, we stated that we examined "indicator loadings, average variance extracted (AVE) values, and composite reliability coefficients" and assessed discriminant validity using "both the Fornell-Larcker criterion and heterotrait-monotrait (HTMT) ratios," but we did not present these values in full. We have now added comprehensive measurement model results to the manuscript. |
| 5. The causal language has not been completely removed after the revision. Please, revise the text again. | We have revised the manuscript to replace all causal language with associational phrasing and added cautious modifiers where appropriate. Changes are highlighted in the resubmitted file. |
