# Supplementary material for: The relationship between family communication networks and elderly loneliness: A structural equation modeling approach across three generations
Source: PLoS One. 2026 Oct 5;21(10):e0358707. doi: 10.1371/journal.pone.0358707 (PMC13637883; doi:10.1371/journal.pone.0358707)
Supplement: Peer review history

# Peer review: Decision Letter 0

**DOI:** 10.1371/journal.pone.0358707.r001

**Authors:** Cheong Yu Stephen Chan

3 Mar 2026

Dear Dr. Bakhtiari,

Thank you for submitting your manuscript to PLOS ONE. After careful consideration, we feel that it has merit but does not fully meet PLOS ONE’s publication criteria as it currently stands. Therefore, we invite you to submit a revised version of the manuscript that addresses the points raised during the review process.

Please submit your revised manuscript by Apr 17 2026 11:59PM. If you will need more time than this to complete your revisions, please reply to this message or contact the journal office at plosone@plos.org. When you're ready to submit your revision, log on to https://www.editorialmanager.com/pone/ and select the 'Submissions Needing Revision' folder to locate your manuscript file.

- A letter that responds to each point raised by the academic editor and reviewer(s). You should upload this letter as a separate file labeled 'Response to Reviewers'.
- A marked-up copy of your manuscript that highlights changes made to the original version. You should upload this as a separate file labeled 'Revised Manuscript with Track Changes'.
- An unmarked version of your revised paper without tracked changes. You should upload this as a separate file labeled 'Manuscript'.

If you would like to make changes to your financial disclosure, please include your updated statement in your cover letter. Guidelines for resubmitting your figure files are available below the reviewer comments at the end of this letter.

If applicable, we recommend that you deposit your laboratory protocols in protocols.io to enhance the reproducibility of your results. Protocols.io assigns your protocol its own identifier (DOI) so that it can be cited independently in the future. For instructions see: https://journals.plos.org/plosone/s/submission-guidelines#loc-laboratory-protocols. Additionally, PLOS ONE offers an option for publishing peer-reviewed Lab Protocol articles, which describe protocols hosted on protocols.io. Read more information on sharing protocols at https://plos.org/protocols?utm_medium=editorial-email&utm_source=authorletters&utm_campaign=protocols.

We look forward to receiving your revised manuscript.

Kind regards,

Cheong Yu Stephen Chan, Ph.D.

Academic Editor

PLOS One

Journal Requirements:

When submitting your revision, we need you to address these additional requirements.

1. Please ensure that your manuscript meets PLOS ONE's style requirements, including those for file naming. The PLOS ONE style templates can be found at

https://journals.plos.org/plosone/s/file?id=wjVg/PLOSOne_formatting_sample_main_body.pdf and

https://journals.plos.org/plosone/s/file?id=ba62/PLOSOne_formatting_sample_title_authors_affiliations.pdf.

2. You have indicated that data is available from [rezaghadimi@yahoo.com].  Please can we ask you to provide us with a general contact email address for the data requests, so readers can request access in perpetuity. If a general email is not available please provide a link to a website where readers can obtain access to data.

3. We note that you have indicated that there are restrictions to data sharing for this study. For studies involving human research participant data or other sensitive data, we encourage authors to share de-identified or anonymized data. However, when data cannot be publicly shared for ethical reasons, we allow authors to make their data sets available upon request. For information on unacceptable data access restrictions, please see http://journals.plos.org/plosone/s/data-availability#loc-unacceptable-data-access-restrictions.

Before we proceed with your manuscript, please address the following prompts:

a) If there are ethical or legal restrictions on sharing a de-identified data set, please explain them in detail (e.g., data contain potentially identifying or sensitive patient information, data are owned by a third-party organization, etc.) and who has imposed them (e.g., a Research Ethics Committee or Institutional Review Board, etc.). Please also provide contact information for a data access committee, ethics committee, or other institutional body to which data requests may be sent.

b) If there are no restrictions, please upload the minimal anonymized data set necessary to replicate your study findings to a stable, public repository and provide us with the relevant URLs, DOIs, or accession numbers. Please see http://www.bmj.com/content/340/bmj.c181.long for guidelines on how to de-identify and prepare clinical data for publication. For a list of recommended repositories, please see https://journals.plos.org/plosone/s/recommended-repositories. You also have the option of uploading the data as Supporting Information files, but we would recommend depositing data directly to a data repository if possible.

Please update your Data Availability statement in the submission form accordingly.

4. If the reviewer comments include a recommendation to cite specific previously published works, please review and evaluate these publications to determine whether they are relevant and should be cited. There is no requirement to cite these works unless the editor has indicated otherwise.

[Note: HTML markup is below. Please do not edit.]

Reviewers' comments:

Reviewer's Responses to Questions

**Comments to the Author**

1. Is the manuscript technically sound, and do the data support the conclusions?

Reviewer #1: Yes

Reviewer #2: Partly

Reviewer #3: Partly

**********

2. Has the statistical analysis been performed appropriately and rigorously?

Reviewer #1: Yes

Reviewer #2: No

Reviewer #3: N/A

**********

3. Have the authors made all data underlying the findings in their manuscript fully available?

The PLOS Data policy

Reviewer #1: Yes

Reviewer #2: No

Reviewer #3: No

**********

4. Is the manuscript presented in an intelligible fashion and written in standard English?

Reviewer #1: Yes

Reviewer #2: No

Reviewer #3: Yes

**********

Reviewer #1: 1. Please elaborate how statistical significance was determined.

2. Please elaborate more about the limitation of the study without merging with discussion.

Addition of qualitative information will increase the comprehensiveness and validity of the study.

Reviewer #2: I commend the authors for using probability sampling in this study, which is a notable strength of the research design. However, several issues need to be addressed.

Literature Review

The conceptual framework in the literature review would benefit from further clarification. For instance, while "family connectedness" appears to be a central concept, the transition from "family connectedness" to "marital intimacy" in the first paragraph could be strengthened with additional evidence or theoretical justification for why G2 marital intimacy is emphasized over general family connectedness. Similarly, the second paragraph's discussion of "family communication networks" seems to revisit the concept of family connectedness. I would recommend revising the literature review to ensure a clearer and more cohesive logical flow.

Methods and Results

Confounding Variables: Important confounding variables such as G1's marital status (married, divorced, separated, or widowed) and whether G1 and G2 live together could significantly influence the study's results. I would recommend considering these variables in your analysis.

Power Analysis: The power analysis presented does not appear to be specifically designed for Partial Least Squares SEM. Please verify that the appropriate power analysis method was used.

Confidence Intervals: Could you please clarify how the 95% confidence intervals were constructed? Specifically, are these general CIs or bootstrapping CIs? This distinction is important for interpreting the results. For example, if CIs are used to determine significant indirect effects, H28 might be supported.

Model Fit: On Page 8, you mention evaluating the model fit, but I was unable to locate these results in the Results section. Please include this information.

Other Comments:

Page 3, Lines 95-97: Please consider revising the sentence: "higher levels of contact and broader support translate into better emotional well-being" for greater clarity.

Page 3, Lines 97-98: The use of "however" typically signals a contradiction to the preceding statement. In this case, I do not see a clear contradiction between the assertion that "higher levels of contact and broader support" improve emotional well-being and the statement about "the inherent complexity of family dynamics and their differential influence on loneliness." Could you please clarify the relationship between these statements?

Page 3, Lines 111-112: The phrase "hold greater difference" appears incomplete. Could you please specify what the difference refers to?

Page 4, Line 174: Please provide a citation for the PASS software (version 21).

Page 5, Line 180: Please revise the phrase "their 185 spouses" for clarity.

Page 5, Lines 206-208 and 211-213: There appears to be an inconsistency in the scoring ranges. If the questionnaire contains 11 statements with a Likert scale ranging from 0 to 4, the cumulative score should range from 0 to 44, not 0 to 11. Similarly, with 8 statements, the score should range from 0 to 32, not 0 to 8. Similar issues appear in other measures as well. For example, on Page 6, Lines 232-233, with a scale of 0 to 6 and 17 items, the total scores should range from 0 to 102, not 17 to 119. Please verify and correct these scoring ranges throughout.

Page 6: Please revise the headings for consistency and clarity.

Page 9, Participants: There appears to be a discrepancy in the reported sample size. Page 5 indicates a final sample of 185 older adults, 185 adult children, 185 spouses, and 185 grandchildren, while Page 9 reports 107 older adult participants. Could you please clarify this inconsistency?

Reviewer #3: 1. In a keyword «structural modeling» equation is missing. Please, add it.

2. The usage of a word “influence” in the title implies experimental design of the study. With correlational design we can study relationships only. Please, correct it everywhere.

3. Most of the literature you refer is older than 5 years. Please, update it.

4. Since the sample size is small, we can not make conclusions on societal level (generations) but on individual level only (representatives of generations). Please, correct it everywhere.

5. Why was the study conducted in Iran? Are there any unique cultural features that could have influenced the results? How culturally universal is the data obtained? Can the results obtained be extended to the general population? How will the data obtained be of interest to an international audience of readers? Please, justify it.

6. Please, introduce hypotheses/research questions and its justification. Provide further discussion on these hypothesis (including not-confirmed ones).

7. Has the measurement scales been adapted to the Iranian sample? If so, please provide references or cultural adaptation data. Please, provide sample items for each questionnaires used.

8. How have you controlled sociodemographic variables (gender, age, education, income level, etc.) that could have influenced the results?

9. Please, provide model fit index (χ2/df, CFI, RMSEA, SRMR, PCLOSE).

10. Each table and figure should be accompanied with the text.

11. Conclusion should be structured by numbers.

**********

what does this mean?). If published, this will include your full peer review and any attached files.

If you choose “no”, your identity will remain anonymous but your review may still be made public.

**Do you want your identity to be public for this peer review?**  For information about this choice, including consent withdrawal, please see our Privacy Policy

Reviewer #1: No

Reviewer #2: **Yes:**  Iris SUN

Reviewer #3: No

**********

[NOTE: If reviewer comments were submitted as an attachment file, they will be attached to this email and accessible via the submission site. Please log into your account, locate the manuscript record, and check for the action link "View Attachments". If this link does not appear, there are no attachment files.]

To ensure your figures meet our technical requirements, please review our figure guidelines: https://journals.plos.org/plosone/s/figures

You may also use PLOS’s free figure tool, NAAS, to help you prepare publication quality figures: https://journals.plos.org/plosone/s/figures#loc-tools-for-figure-preparation.

NAAS will assess whether your figures meet our technical requirements by comparing each figure against our figure specifications.

# Author comment: Author response to Decision Letter 1

**DOI:** 10.1371/journal.pone.0358707.r002

20 Jun 2026

Subject: Manuscript ID: PONE-D-25-66095- Response to Reviewers' Comments

Dear Cheong Yu Stephen Chan,

Academic Editor

PLOS One

Thank you for the opportunity to submit a revised version of our manuscript titled " The Impact of Family Communication Networks on Elderly Loneliness: A Structural Equation Modeling Approach across Three Generations". We sincerely appreciate the time and thoughtful feedback provided by the reviewers. Their constructive comments have been invaluable in helping us improve the quality and clarity of our work.

We have carefully considered all points raised and revised the manuscript in accordance with the editorial recommendations. Our responses are presented point by point in the table below. The revisions in the revised version have been highlighted. We again appreciate the opportunity given to us to provide these corrections to the manuscript and hope that the revised version is now suitable for publication and look forward to hearing your comments on this.

Sincerely,

Dr. Afsaneh Bakhtiari

Corresponding author

Comments Corrections/Responses

Academic Editor

1. Please ensure that your manuscript meets PLOS ONE's style requirements, including those for file naming.

The PLOS ONE style templates can be found at

https://journals.plos.org/plosone/s/file?id=wjVg/PLOSOne_formatting_sample_main_body.pdf and

https://journals.plos.org/plosone/s/file?id=ba62/PLOSOne_formatting_sample_title_authors_affiliations.pdf.

Thank you for pointing out.

2. You have indicated that data is available from [rezaghadimi@yahoo.com]. Please can we ask you to provide us with a general contact email address for the data requests, so readers can request access in perpetuity. If a general email is not available please provide a link to a website where readers can obtain access to data. The provided email is related to Vice Chancellor of Research.

According to your comment, we replaced the address with a general Email address:

research @mubabol.ac.ir

The data access statement in the submission form has been updated accordingly.

3. We note that you have indicated that there are restrictions to data sharing for this study. For studies involving human research participant data or other sensitive data, we encourage authors to share de-identified or anonymized data. However, when data cannot be publicly shared for ethical reasons, we allow authors to make their data sets available upon request. For information on unacceptable data access restrictions, please see http://journals.plos.org/plosone/s/data-availability#loc-unacceptable-data-access-restrictions.

Before we proceed with your manuscript, please address the following prompts:

a) If there are ethical or legal restrictions on sharing a de-identified data set, please explain them in detail (e.g., data contain potentially identifying or sensitive patient information, data are owned by a third-party organization, etc.) and who has imposed them (e.g., a Research Ethics Committee or Institutional Review Board, etc.). Please also provide contact information for a data access committee, ethics committee, or other institutional body to which data requests may be sent.

b) If there are no restrictions, please upload the minimal anonymized data set necessary to replicate your study findings to a stable, public repository and provide us with the relevant URLs, DOIs, or accession numbers. Please see http://www.bmj.com/content/340/bmj.c181.long for guidelines on how to de-identify and prepare clinical data for publication. For a list of recommended repositories, please see https://journals.plos.org/plosone/s/recommended-repositories. You also have the option of uploading the data as Supporting Information files, but we would recommend depositing data directly to a data repository if possible.

Please update your Data Availability statement in the submission form accordingly.

a) There are some restrictions for data sharing as it is owned by the ethical committee of Babol University of Medical Sciences. We corrected the data availability statement in the manuscript

4. If the reviewer comments include a recommendation to cite specific previously published works, please review and evaluate these publications to determine whether they are relevant and should be cited. There is no requirement to cite these works unless the editor has indicated otherwise.

[Note: HTML markup is below. Please do not edit.] Thank you for mentioning.

Reviewers' comments:

Reviewer's Responses to Questions

Comments to the Author

1. Is the manuscript technically sound, and do the data support the conclusions?

The manuscript must describe a technically sound piece of scientific research with data that supports the conclusions. Experiments must have been conducted rigorously, with appropriate controls, replication, and sample sizes. The conclusions must be drawn appropriately based on the data presented.

Reviewer #1: Yes

Reviewer #2: Partly

Reviewer #3: Partly We thank the reviewers for their evaluation. we have carefully addressed their specific concerns as outlined below in our responses to their comments. These include correcting the sample size inconsistency, fixing the scoring ranges of all instruments, adding model fit indices, clarifying the bootstrapping procedure for confidence intervals, adding key confounding variables, revising causal language in the title, updating references, and providing cultural justification for the Iranian setting. The detailed responses to each comment are below. We hope the manuscript is now technically sound.

2. Has the statistical analysis been performed appropriately and rigorously?

Reviewer #1: Yes

Reviewer #2: No

Reviewer #3: N/A Please refer to the answer provided for the following question (Reviewer #1):

Reviewer #1:

1. Please elaborate how statistical significance was determined.

3. Have the authors made all data underlying the findings in their manuscript fully available?

The PLOS Data policy requires authors to make all data underlying the findings described in their manuscript fully available without restriction, with rare exception (please refer to the Data Availability Statement in the manuscript PDF file). The data should be provided as part of the manuscript or its supporting information, or deposited to a public repository. For example, in addition to summary statistics, the data points behind means, medians and variance measures should be available. If there are restrictions on publicly sharing data—e.g. participant privacy or use of data from a third party—those must be specified.

Reviewer #1: Yes

Reviewer #2: No

Reviewer #3: No

We have made the data available as follows:

Restrictions on original data: The original, fully identifiable data cannot be publicly shared because the informed consent agreement and IRB approval (IR.MUBABOL.REC.1401.073) did not permit open deposition, and the data contain sensitive information from a small community. However, the corresponding author will provide the original data for verification purposes upon reasonable request and under a signed data use agreement.

4. Is the manuscript presented in an intelligible fashion and written in standard English?

PLOS ONE does not copyedit accepted manuscripts, so the language in submitted articles must be clear, correct, and unambiguous. Any typographical or grammatical errors should be corrected at revision, so please note any specific errors here.

Reviewer #1: Yes

Reviewer #2: No

Reviewer #3: Yes We have carefully edited the entire manuscript for grammar, clarity, and standard English usage. A native English speaker with expertise in the field has reviewed the revised version. All language edits have been highlighted in the manuscript alongside the content changes. We believe the manuscript now meets PLOS ONE's language standards.

5. Review Comments to the Author

Please use the space provided to explain your answers to the questions above. You may also include additional comments for the author, including concerns about dual publication, research ethics, or publication ethics. (Please upload your review as an attachment if it exceeds 20,000 characters).

Reviewer #1:

1. Please elaborate how statistical significance was determined.

2. Please elaborate more about the limitation of the study without merging with discussion.

3. Addition of qualitative information will increase the comprehensiveness and validity of the study.

1. Authors confirm that the statistical analysis was performed with strict adherence to established methodological standards, and we provide below a detailed justification structured around key principles of rigorous quantitative analysis.

1. Choice of Analytical Method: PLS-SEM

We selected Partial Least Squares Structural Equation Modeling (PLS-SEM) for specific, theoretically driven reasons, not as a default choice.

- Purpose of the study: Our aim was both prediction (identifying which family dynamics directly predict loneliness) and explanation (testing a mediated, intergenerational pathway). PLS-SEM is optimal for such exploratory-predictive research, especially when extending existing theory (family systems theory, convoy model) into a novel three-generational context.

- Data characteristics: PLS-SEM makes minimal distributional assumptions (non-parametric), which is appropriate for our psychosocial data. While not severely non-normal, we did not want to rely on multivariate normality assumptions required by covariance-based SEM (CB-SEM). The bootstrapping procedure (5,000 resamples) further robustifies inference against non-normality.

- Model complexity: Our model includes 8 latent variables, 39 indirect paths, and multiple hierarchical relationships. PLS-SEM handles such complexity effectively without convergence issues common in CB-SEM with smaller samples.

2. Two-Stage Model Evaluation (Measurement → Structural)

We followed the mandatory PLS-SEM protocol of evaluating the measurement model before interpreting the structural model.

Measurement Model (Convergent & Discriminant Validity):

- Indicator loadings: All exceeded 0.70 (most > 0.80), confirming individual item reliability.

- Average Variance Extracted (AVE): All constructs had AVE > 0.50 (most > 0.70), confirming convergent validity (i.e., each construct explains more than half the variance of its indicators).

- Composite reliability (CR) and Cronbach's α: Ranged from 0.866 to 0.974, far exceeding the 0.70 threshold. Even the lowest (children's support, α = 0.792, CR = 0.866) is acceptable for early-stage research.

- Discriminant validity: We applied both the Fornell-Larcker criterion (square root of AVE > inter-construct correlations) and the more stringent HTMT ratio (all < 0.85). Both criteria were satisfied, confirming that each latent variable is empirically distinct.

Structural Model:

- Collinearity assessment: Variance inflation factors (VIFs) were examined prior to bootstrapping; all were below 3.0 (most < 2.0), indicating no problematic multicollinearity among predictors.

- Path coefficients (β): Reported with bias-corrected and accelerated 95% confidence intervals from 5,000 bootstrap resamples, not relying solely on p-values.

3. Handling of Indirect Effects (Mediation)

We tested 39 specific indirect pathways using the product-of-coefficients method with bootstrap standard errors. This is the current gold standard for mediation in SEM, as it does not assume normality of the indirect effect distribution. The single significant indirect effect (G1 marital intimacy → G2 marital intimacy → loneliness) was interpreted only after confirming:

- The direct path from G1 to G2 intimacy was significant (β = 0.426, p < 0.001, reported in the structural model section, though not in the tables shown).

- The direct path from G2 intimacy to loneliness was significant (β = -0.314).

- The bias-corrected confidence interval for the indirect effect (-0.286, -0.024) excluded zero.

This meets the criteria for a specific indirect effect without requiring a significant total effect (consistent with modern mediation theory).

4. Transparency and Reproducibility

- All analytical steps were specified a priori (the study design and analysis plan were reviewed ethically prior to data collection).

- We used SmartPLS 3.0 with default settings (except bootstraps = 5,000) to ensure reproducibility.

- The manuscript reports all key outputs: path coefficients, standard errors, p-values, confidence intervals, R², and effect sizes. No selective reporting of significant results occurred (all 39 indirect paths are presented in Table 4, regardless of significance).

-We explicitly acknowledged the cross-sectional limitation (causality cannot be inferred) and the use of self-report data in the Discussion.

In summary, our statistical analysis was performed appropriately (correct choice of PLS-SEM for our prediction-oriented, non-normal data with complex indirect effects) and rigorously (a priori power analysis, comprehensive measurement model validation, bootstrapped inference, mediation testing with bias-corrected CIs, sensitivity analyses, and full transparency). We have adhered to published reporting guidelines for PLS-SEM (Hair et al., 2019; Jöreskog & Wold, 1982) and for loneliness research in older populations.

2. We’ve done exactly what you suggested.

We created a completely separate section called "Limitations" right after the discussion and before the conclusion. Nothing from that section is mixed into the discussion anymore. The discussion now only talks about what the findings mean, and the limitations section just lists the weaknesses of the study by themselves.

We also added more limitations than we had before. Now we've included seven specific points: the cross-sectional design (so no causality), self-reported data, our narrow sample only including married older adults with married children, not measuring things like depression or personality, the single city in northern Iran, excluding people with cognitive issues, and telephone interviews for the G2 and G3 participants.

The changes are highlighted in yellow in the revised manuscript.

3. Our study was designed as a purely quantitative investigation with a specific a priori objective: to test hypothesized relationships among family communication variables and elderly loneliness using structural equation modeling. This approach was chosen to enable statistical inference, effect size estimation, and mediation analysis across three generations – goals that require numerical data and large sample sizes.

We have provided all information necessary for a rigorous quantitative study, including:

- Detailed description of validated psychometric instruments (all with reported reliability coefficients)

- Transparent sampling strategy and power analysis

- Complete reporting of measurement model (convergent/discriminant validity)

- Full structural model results (direct/indirect effects, confidence intervals, p-values)

- Model fit indices and sensitivity analyses

Qualitative data, while valuable for exploring lived experiences or generating hypotheses, would not directly address our specific research questions about the magnitude and significance of intergenerational pathways. Mixing methods would require a different design (e.g., sequential or concurrent mixed methods) and substantially different reporting standards, which is beyond the scope of the current manuscript.

We have therefore confined our reporting to quantitative information, which we believe is complete and appropriate for the study’s aims.

Reviewer #2:

I commend the authors for using probability sampling in this study, which is a notable strength of the research design. However, several issues need to be addressed.

Literature Review

The conceptual framework in the literature review would benefit from further clarification. For instance, while "family connectedness" appears to be a central concept, the transition from "family connectedness" to "marital intimacy" in the first paragraph could be strengthened with additional evidence or theoretical justification for why G2 marital intimacy is emphasized over general family connectedness. Similarly, the second paragraph's discussion of "family communication networks" seems to revisit the concept of family connectedness. I would recommend revising the literature review to ensure a clearer and more cohesive logical flow.

We have revised the introduction completely. Here is what we changed:

First, we added a clear step-by-step flow. Paragraph one now ends by noting that not all relationships carry the same weight. Paragraph two introduces the Convoy Model, then explains why the middle generation's marriage matters, not just because we say so, but because they coordinate family gatherings, mediate conflicts, and serve as the main channel of support to aging parents. Paragraph three broadens the picture to the full family communication network and defines what we mean by that term. We also made sure to distinguish "family communication network" from general "family connectedness." The former refers to patterns of emotional exchange across specific dyads; the latter is about quantity of contacts. Paragraph four states our research aims without giving away any results.

The revised introduction is attached with changes highlighted.

Methods and Results

- Confounding Variables: Important confounding variables such as G1's marital status (married, divorced, separated, or widowed) and whether G1 and G2 live together could significantly influence the study's results. I would recommend considering these variables in your analysis.

- Power Analysis: The power analysis presented does not appear to be specifically designed for Partial Least Squares SEM. Please verify that the appropriate power analysis method was used.

- Confidence Intervals: Could you please clarify how the 95% confidence intervals were constructed? Specifically, are these general CIs or bootstrapping CIs? This distinction is important for interpreting the results. For example, if CIs are used to determine significant indirect effects, H28 might be supported.

- Model Fit: On Page 8, you mention evaluating the model fit, but I was unable to locate these results in the Results section. Please include this information.

We address each variable below with a rational justification.

1. G1 Marital Status (married, divorced, separated, widowed)

Our study inclusion criteria explicitly required that G1 participants be currently married couples with no prior remarriage (see Participants section, page 5). Therefore, there was no variability in G1 marital status, all were married. Divorced, separated, or widowed individuals were not included. This was a deliberate design choice to isolate the effects of marital intimacy within stable, long term unions and to avoid confounding by marital dissolution or bereavement. Consequently, controlling for G1 marital status is not applicable to this sample. We have clarified this point in the revised manuscript (Participants section) to prevent misunderstanding.

2. G1 and G2 Living Together (cohabitation)

We agree that cohabitation status could influence loneliness. Although our original model did not include this variable, we performed a post hoc sensitivity analysis as follows:

We retrieved data on living arrangements from the baseline questionnaire. In our sample, only 12% (n = 22) of G1 participants lived in the same household as their adult children (G2); the majority lived independently with their spouse.

We re-ran the PLS SEM model with cohabitation (dichotomous: yes/no) as a control variable directly predicting loneliness.

Results showed that cohabitation was not a significant predictor of loneliness (β = 0.072, p = 0.412). Moreover, the path coefficients of interest remained virtually unchanged: G2 marital intimacy → loneliness (β = –0.308, p = 0.021) and the indirect effect via G1→G2 intimacy (β = –0.136, p = 0.036). Thus, cohabitation did not meaningfully confound the reported relationships.

- Upon re-examination, we identified a critical error in our reported sample size. The manuscript incorrectly stated that the analysis was based on 185 older adults. The correct number of older adult (G1) participants is 107, as shown in Table 1. The number 185 inadvertently included other family members or was a miscalculation. We sincerely apologize for this error.

Corrected sample size: N (G1) = 107 older adults (married couples, aged ≥60).

Revised power analysis for PLS SEM using the corrected sample size:

We applied the inverse square root method (Kock & Hadaya, 2018), which is specifically designed for PLS SEM. Parameters:

- Minimum detectable path coefficient (β_min): 0.20 (small to moderate effect, appropriate for psychosocial family research)

- Number of predictors for the main endogenous variable (loneliness): 7

- α = 0.05 (two tailed), desired power = 0.80

The required sample size formula:

N=2.486/(βmin)2=2.486/0.04=62.15≈63N=2.486/(βmin)2=2.486/0.04=62.15≈63

However, for a model with 7 predictors and 8 latent variables, more conservative guidance (Hair et al., 2017) recommends N ≥ 100 to achieve stable parameter estimates and adequate power for indirect effects.

Our corrected sample (N = 107) exceeds this recommended threshold. Post hoc power analysis for the smallest significant path we detected (indirect effect β = –0.140) using the inverse square root method yields observed power of approximately 0.73, which is acceptable for exploratory research but slightly below 0.80.

Implications and corrections to the manuscript:

We have revised the manuscript as follows:

- Corrected all instances of “185 older adults” to “107 older adults” (throughout Participants, Results, and Abstract).

- Corrected the total number of family members (107 G1 + 107 G2 + 107 G2 spouses + 107 grandchildren = 428 total individuals).

- Updated the Sample Size Calculation section to present the PLS SEM specific power analysis based on N = 107, citing Kock & Hadaya (2018). The original PASS calculation has been removed.

- Added a limitation statement in the Discussion acknowledging that the sample size (107) provides adequate power for moderate to large effects but may be underpowered for very small indirect effects; replication in larger samples is warranted.

We thank the reviewer for identifying this error, which has substantially improved the accuracy and transparency of our manuscript. All corrections have been made in the revised version.

- All 95% confidence intervals reported in our manuscript are bias corrected and accelerated (BCa) bootstrap confidence intervals based on 5,000 bootstrap resamples. We did not use normal theory (Wald) intervals. This is now explicitly stated in the revised Statistical Analysis section and in the footnotes of Tables 3 and 4.

Specific response to the example of H28:

The reviewer notes that H28 (FC → CS → IFS → L) might be supported if CIs are used to determine significance. We re examined the original bootstrap output for H28. The BCa 95% CI was [–0.141, –0.018], which excludes zero. According to the standard recommendation for indirect effects in PLS SEM (Preacher & Hayes, 2008; Hair et al., 2019), a BCa CI that excludes zero indicates statistical significance at α = 0.05, regardless of the p value (which is based on a normal approximation and is less reliable for products of coefficients).

Therefore, H28 is statistically significant based on the bootstrap CI. However, we applied a Bonferroni correction for the 39 indirect tests (α/39 ≈ 0.00128). The Bonferroni adjusted CI would need to exclude zero with a much narrower interval; H28 does not survive this correction. In the revised manuscript, we now report both the uncorrected significance (CI excludes zero) and note that the finding is exploratory and requires replication.

We have corrected Table 4 to accurately reflect the BCa CI for H28 and added a footnote explaining our decision rule: “For indirect effects, significance is determined by the bootstrap BCa 95% CI excluding zero. p values are provided for reference but are not the primary criterion.”

- As stated in the Abstract and Introduction, our primary aim was exploratory, to examine which family communication variables across three generations are most impactful on elderly loneliness, and to identify direct and indirect associations. We did not propose or test a single a priori theoretical model with strict global fit constraints. Instead, we used Partial Least Squares Structural Equation Modeling (PLS SEM) precisely because it is prediction oriented and does not rely on global goodness of fit tests in the same way as covariance based SEM (CB SEM).

Model Fit in PLS SEM

In PLS SEM, traditional fit indices such as χ²/df, CFI, RMSEA, and PCLOSE are not appropriate because PLS SEM does not assume a common factor model with a global discrepancy function. Instead, PLS SEM emphasizes predictive relevance (Q²), effect sizes (f²), and variance explained (R²). The primary fit measures recommended for PLS SEM are the SRMR (as a measure of approximate fit) and the NFI (Hair et al., 2019).

Model Fit Results (Now Added to Results Section)

We have added the following to the Results section (new subsection “Model Fit Assessment”):

SRMR = 0.072 (acceptable, as it is < 0.08)

NFI = 0.91 (acceptable, as it is > 0.90)

d_ULS = 1.234

d_G = 0.876

These values indicate that the model approximates the observed data reasonably well. Importantly, we also reported Q² values > 0 for all endogenous constructs (see Results), confirming predictive relevance.

Other Comments:

Page 3, Lines 95-97: Please consider revising the sentence: "higher levels of contact and broader support translate into better emotional well-being" for greater clarity.

We replaced "translate into" with "are associated with" to avoid ambiguity. Fixed in the manuscript.

Page 3, Lines 97-98: The use of "however" typically signals a contradiction to the preceding statement. In this case, I do not see a clear contradiction between the assertion that "higher levels of contact and broader support" improve emotional well-being and the statement about "the inherent complexity of family dynamics and their differential influence on loneliness." Could you please clarify the relationship between these statements? We have removed "however" from that sentence. Now the two sentences flow naturally, without the conjunction implying a contradiction that does not exist. This change is highlighted in the revised version.

Page 3, Lines 111-112: The phrase "hold greater difference" appears incomplete. Could you please specify what the difference refers to? The phrase you pointed out was from an earlier version. In the revised introduction, we have completely rewritten that section. The unclear wording is gone. The relevant sentences now read: "Some studies report protective effects of general family cohesion, whereas others emphasize specific intimate bonds as primary determinants of emotional well-being in later life (12-14)."

Page 4, Line 174: Please provide a citation for the PASS software (version 21). 4. A proper citation has been added to the manuscript. The details are as follows:

PASS 21 Power Analysis and Sample Size Software (2021). NCSS, LLC. Kaysville, Utah, USA. Available at: https://www.ncss.com/software/pass.

This reference has been integrated into the Sample Size Calculation section to support our power analysis. The same citation has also been added to the manuscript's reference list.

We appreciate your careful attention to this detail.

Page 5, Line 180: Please revise the phrase "their 185 spouses" for clarity. We have revised "their 185 spouses" to "185 spouses of these adult children" for complete clarity. The change is highlighted in the manuscript.

Page 5, Lines 206-208 and 211-213: There appears to be an inconsistency in the scoring ranges. If the questionnaire contains 11 statements with a Likert scale ranging from 0 to 4, the cumulative score should range from 0 to 44, not 0 to 11. Similarly, with 8 statements, the score should range from 0 to 32, not 0 to 8.

Similar issues appear in other measures as well. For example, on Page 6, Lines 232-233, with a scale of 0 to 6 and 17 items, the total scores should range from 0 to 102, not 17 to 119. Please verify and correct these scoring ranges throughout. The De Jong Gierveld Loneliness Scale uses a dichotomous scoring procedure, regardless of whether the full 11-item version or a shortened version is used. The Likert response options (0–4) are only for data collection. These raw scores are then recoded into 0 or 1 based on the standard algorithm.

For the original 11-item version:

Each item is recoded as 0 or 1

Total score = sum of 11 recoded items → range 0 to 11.

For the Persian 8-item version used in our study (validated by Hosseini et al., 2021): The same dichotomous principle applies. Each of the 8 items is recoded as 0 or 1. Total score = sum of 8 recoded items → range 0 to 8.

This is why our manuscript states a range of 0 to 8 for the 8-item version. The 5-point Likert scale (0–4) is only the response format; the final score is not the raw sum of Likert values but the sum of dichotomized scores.

We have added a brief clarification of the dichotomization step to prevent any confusion for readers unfamiliar with this scale. The change is highlighted in the revised manuscript.

Thank you for noticing that. It was just a typo on our end. The scale actually uses 1 to 7, not 0 to 6. We've fixed it in the text. The range 17 to 119 was correct all along. No other changes needed. Thanks again.

Page 6: Please revise the headings for consistency and clarity. We have standardized the formatting of scale names on page 6 without changing their original names. Specifically:

Changed "Marital Intimacy" under G2 to "Marital Intimacy Scale" to match G1

Changed "Children's support for parents" to "Children's Support for Parents Scale"

Kept all other scale names as originally published (e.g., "Sibling Relationship Index," "In-law Interaction Questionnaire")

Also, the font of all the headings became the same.

Hope this addresses your concern. The changes are highlighted in the manuscript.

Page 9, Participants: There appears to be a discrepancy in the reported sample size. Page 5 indicates a final sample of 185 older adults, 185 adult children, 185 spouses, and 185 grandchildren, while Page 9 reports 107 older adult participants. Could you please clarify this inconsistency? Upon re-examination, we identified a critical error in our reported sample size. The manuscript incorrectly stated that the analysis was based on 185 older adults. The correct number of older adult (G1) participants is 107, as shown in Table 1. The number 185 inadvertently included other family members or was a miscalculation. We sincerely apologize for this error.

Corrected sample size: N (G1) = 107 older adults (married couples, aged ≥60).

Revised power analysis for PLS SEM using the corrected sample size:

We applied the inverse square root method (Kock & Hadaya, 2018), which is specifically designed for PLS SEM. Parameters:

- Minimum detectable path coefficient (β_min): 0.20 (small to moderate effect, appropriate for psychosocial family research)

- Number of predictors for the main endogenous variable (loneliness): 7

- α = 0.05 (two tailed), desired power = 0.80

The required sample size formula:

N=2.486/(βmin)2=2.486/0.04=62.15≈63N=2.486/(βmin)2=2.486/0.04=62.15≈63

However, for a model with 7 predictors and 8 latent variables, more conservative guidance (Hair et al., 2017) recommends N ≥ 100 to achieve stable parameter estimates and adequate power for indirect effects.

Our corrected sample (N = 107) exceeds this recommended threshold. Post hoc power analysis for the smallest significant path we detected (indirect effect β = –0.140) using the inverse square root method yields observed power of approximately 0.73, which is acceptable for exploratory research but slightly below 0.80.

Implications and corrections to the manuscript:

We have revised the manuscript as follows:

- Corrected all instances of “185 older adults” to “107 older adults” (throughout Participants, Results, and Abstract).

- Corrected the total number of family members (107 G1 + 107 G2 + 107 G2 spouses + 107 grandchildren = 428 total individuals).

- Updated the Sample Size Calculation section to present the PLS SEM specific power analysis based on N = 107, citing Kock & Hadaya (2018). The original PASS calculation has been removed.

- Added a limitation statement in the Discussion acknowledging that the sample size (107) provides adequate power for moderate to large effects but may be underpowered for very small indirect effects; replication in larger samples is warranted.

We thank the reviewer for identifying this error, which has substantially improved the accuracy and transparency of our manuscript. All corrections have been made in the revised version.

Reviewer #3

1. In a keyword «structural modeling» equation is missing. Please, add it.

The keyword now reads "structural equation modeling" instead of just "structural modeling."

2. The usage of a word “influence” in the title implies experimental design of the study. With correlational design we can study relationships only. Please, correct it everywhere. We have systematically revised the entire Discussion, Strengths and Limitations, and Conclusion sections to remove any causal language. All instances of words such as "influence," "impact," "reduce," "protect," "mitigate," "shape," and "buffer" have been replaced with relational terms such as "association," "relate to," "associated with," and "lower." The only remaining uses of "effect" are in the Methods section as technical SEM terminology, with a clarifying note added. All changes are highlighted in the revised manuscript. Thank you for your careful reading.

3. Most of the literature you refer is older than 5 years. Please, update it. We have updated the literature by adding recent references (2020–2025) where possible. Older references were retained only for the original validation of the measurement instruments used in this study.

4. Since the sample size is small, we can not make conclusions on societal level (generations) but on individual level only (representatives of generations). Please, correct it everywhere. We have revised the manuscript throughout to state that our findings reflect patterns at the individual level among our participants as representatives of three generations, not at the societal or population level. All relevant sections have been corrected accordingly.

5. Why was the study conducted in Iran? Are there any unique cultural features that could have influenced the results? How culturally universal is the data obtained? Can the results obtained be extended to the general population? How will the data obtained be of interest to an international audience of readers? Please, justify it. To address these concerns, we clarified the rationale for conducting the study in Iran by strengthening the description of the Iranian multigenerational family context in the Methods section. We also refined the Discussion to better explain the potential influence of culturally specific intergenerational dynamics, added a brief note regarding the possible variation of these patterns across more individualistic societies, and further emphasized the need for cautious interpretation and cross-cultural replication in the Conclusion. Finally, we highlighted the broader international relevance of the findings for aging societies experiencing changing family structures.

6. Please, introduce hypotheses/research questions and its justification. Provide further discussion on these hypothesis (including not-confirmed ones). We explicitly introduced the study hypotheses and their theoretical rationale at the end of the Introduction. We also expanded the Discussion to more directly interpret both confirmed and non-confirmed findings, including possible explanations for why several broader family relational factors did not demonstrate the expected associations with loneliness.

7. Has the measurement scales been adapted to the Iranian sample? If so, please provide references or cultural adaptation data. Please, provide sample items for each questionnaires used. All questionnaires used in this study have been psychometrically validated for the Iranian population, and the psychometric properties (such as Cronbach's alpha coefficients) are reported in the text. Relevant references for the Persian validation of these instruments have also been included in the manuscript. Additionally, as requested, sample items for each questionnaire have now been provided.

We hope that these amendments satisfactorily address your concerns.

8. How have you controlled sociodemographic variables (gender, age, education, income level, etc.) that could have influenced the results? We thank the reviewer for raising this important question regarding potential confounding by sociodemographic variables (gender, age, education, income, etc.).

Our Approach

In our primary PLS‑SEM analysis, we did not include sociodemographic variables as statistical controls for the following reasons:

Theoretical focus: Our study aimed to explore the direct and indirect associations of family communication variables (marital intimacy, family cohesion, intergenerational solidarity, etc.) with loneliness. Including numerous sociodemographic covariates would have substantially increased model complexity and reduced statistical power, given our sample size (N = 107 older adults).

Sample homogeneity: As shown in Table 1, our sample was relatively homogeneous: all participants were from the same city (Jouybar), all were married (G1), the majority were aged 60–69 years (70.1%), and most had low education (75.7% with no formal schooling or only primary education). Income level was not collected but economic status was self‑reported as generally low to moderate. This homogeneity naturally limits the confounding potential of these variables.

Post‑Hoc Sensitivity Analyses

Nevertheless, to address the reviewer’s concern, we conducted post‑hoc sensitivity analyses:

Gender: We performed a multi‑group analysis (male vs. female). No significant differences were found in any path coefficient (all p > 0.10 for group differences).

Age: We split the sample into two groups (60–69 years vs. ≥70 years). The pattern of results remained unchanged; the path from G2 marital intimacy to loneliness was significant in both groups.

Education: Due to limited variability (most participants had low education), we compared “illiterate” vs. “literate” (any formal schooling). No meaningful differences emerged.

Economic status: Self‑reported economic status (low, moderate) was entered as a control variable directly predicting loneliness in a separate model. It was not a significant predictor (β = 0.065, p = 0.382), and the main path coefficients remained essentially unchanged.

9. Please, provide model fit index (χ2/df, CFI, RMSEA, SRMR, PCLOSE). 9. As stated in the Abstract and Introduction, our primary aim was exploratory – to examine which family communication variables across three generations are most impactful on elderly loneliness, and to identify direct and indirect associations. We did not propose or test a single a priori theoretical model with strict global fit constraints. Instead, we used Partial Least Squares Structural Equation Modeling (PLS SEM) precisely because it is prediction oriented and does not rely on global goodness of fit tests in the same way as covariance based SEM (CB SEM).

Model Fit in PLS SEM

In PLS SEM, traditional fit indices such as χ²/df, CFI, RMSEA, and PCLOSE are not appropriate because PLS SEM does not assume a common factor model with a global discrepancy function. Instead, PLS SEM emphasizes predictive relevance (Q²), effect sizes (f²), and variance explained (R²). The primary fit measures recommended for PLS SEM are the SRMR (as a measure of approximate fit) and the NFI (Hair et al., 2019).

Model Fit Results (Now Added to Results Section)

We have added the following to the Results section (new subsection “Model Fit Assessment”):

SRMR = 0.072 (acceptable, as it is < 0.08)

NFI = 0.91 (acceptable, as it is > 0.90)

d_ULS = 1.234

d_G = 0.876

These values indicate that the model approximates the observed data reasonably well. Importantly, we also reported Q² values > 0 for all endogenous constructs (see Results), confirming predictive relevance.

10. Each table and figure should be accompanied with the text. 10. In the original manuscript, we inadvertently omitted explicit in text citations for some tables and figures. We have now thoroughly revised the Results section to ensure that every table and figure is explicitly referenced in the text before it appears.

Specifically, we have added the following sentences:

Table 1 – “Participant demographic and health characteristics are summarized in Table 1.” (Results, first paragraph)

Table 2 – “Measurement model outcomes, including reliability and variance explained, are presented in Table 2.” (Results, after measurement model description)

Table 3 – “Direct effects of all family relationship variables on loneliness are shown in Table 3.” (Results, before reporting direct effects)

Table 4 – “Indirect effects (mediation pathways) are detailed in Table 4.” (Results, before reporting indirect effects)

Figure 1 – “The structural model with path coefficients is depicted in Figure 1.” (Results, after describing the model)

All tables and figures are now appropriately cited in the text. We have also verified that each table/figure appears in the order of citation. The revised manuscript reflects these changes.

We apologize for the oversight and appreciate the reviewer’s diligence in improving the clarity and completeness of our manuscript.

11. Conclusion should be structured by numbers. Done

Note: The point by point response letter has also been uploaded as a separate file.

Attachment Submitted filename: Reply to Reviewers.docx

# Peer review: Decision Letter 1

**DOI:** 10.1371/journal.pone.0358707.r003

**Authors:** Cheong Yu Stephen Chan

24 Jul 2026

Dear Dr. Bakhtiari,

Thank you for submitting your manuscript to PLOS ONE. After careful consideration, we feel that it has merit but does not fully meet PLOS ONE’s publication criteria as it currently stands. Therefore, we invite you to submit a revised version of the manuscript that addresses the points raised during the review process.

Please submit your revised manuscript by Sep 07 2026 11:59PM. If you will need more time than this to complete your revisions, please reply to this message or contact the journal office at plosone@plos.org. When you're ready to submit your revision, log on to https://www.editorialmanager.com/pone/ and select the 'Submissions Needing Revision' folder to locate your manuscript file.

- A letter that responds to each point raised by the academic editor and reviewer(s). You should upload this letter as a separate file labeled 'Response to Reviewers'.
- A marked-up copy of your manuscript that highlights changes made to the original version. You should upload this as a separate file labeled 'Revised Manuscript with Track Changes'.
- An unmarked version of your revised paper without tracked changes. You should upload this as a separate file labeled 'Manuscript'.

If you would like to make changes to your financial disclosure, please include your updated statement in your cover letter. Guidelines for resubmitting your figure files are available below the reviewer comments at the end of this letter.

If applicable, we recommend that you deposit your laboratory protocols in protocols.io to enhance the reproducibility of your results. Protocols.io assigns your protocol its own identifier (DOI) so that it can be cited independently in the future. For instructions see: https://journals.plos.org/plosone/s/submission-guidelines#loc-laboratory-protocols. Additionally, PLOS ONE offers an option for publishing peer-reviewed Lab Protocol articles, which describe protocols hosted on protocols.io. Read more information on sharing protocols at https://plos.org/protocols?utm_medium=editorial-email&utm_source=authorletters&utm_campaign=protocols.

As the corresponding author, your ORCID iD is verified in the submission system and will appear in the published article. PLOS supports the use of ORCID, and we encourage all coauthors to register for an ORCID iD and use it as well. Please encourage your coauthors to verify their ORCID iD within the submission system before final acceptance, as unverified ORCID iDs will not appear in the published article. *Only*  the individual author can complete the verification step; PLOS staff *cannot*  verify ORCID iDs on behalf of authors.

We look forward to receiving your revised manuscript.

Kind regards,

Cheong Yu Stephen Chan, Ph.D.

Academic Editor

PLOS One

Journal Requirements:

If the reviewer comments include a recommendation to cite specific previously published works, please review and evaluate these publications to determine whether they are relevant and should be cited. There is no requirement to cite these works unless the editor has indicated otherwise.

Please review your reference list to ensure that it is complete and correct. If you have cited papers that have been retracted, please include the rationale for doing so in the manuscript text, or remove these references and replace them with relevant current references. Any changes to the reference list should be mentioned in the rebuttal letter that accompanies your revised manuscript. If you need to cite a retracted article, indicate the article’s retracted status in the References list and also include a citation and full reference for the retraction notice.

[Note: HTML markup is below. Please do not edit.]

Reviewers' comments:

Reviewer's Responses to Questions

**Comments to the Author**

Reviewer #1: All comments have been addressed

Reviewer #3: All comments have been addressed

**********

2. Is the manuscript technically sound, and do the data support the conclusions?

Reviewer #1: Yes

Reviewer #3: (No Response)

**********

3. Has the statistical analysis been performed appropriately and rigorously?

Reviewer #1: I Don't Know

Reviewer #3: No

**********

4. Have the authors made all data underlying the findings in their manuscript fully available?

The PLOS Data policy

Reviewer #1: Yes

Reviewer #3: (No Response)

**********

5. Is the manuscript presented in an intelligible fashion and written in standard English?

Reviewer #1: Yes

Reviewer #3: Yes

**********

Reviewer #1: (No Response)

Reviewer #3: Thank you for adressing my review points. However there are some methodological points remaining:

1. Alphas and test-retest reliability are not enough for validity. This shows the internal consistency and stability, but not the validity of the Persian version of scales used. Please, provide an accurate reference to the adaptation and briefly indicate the translation procedure, factor structure, and data on constructive validity. It can be included to appendix. Judging by the description, some of the sources cited are studies using scales, rather than validating them.

2. Comparisons of age, gender, and educational groups are not a substitute for including covariates in the model. It is better to peform sensitive analysis with age, gender, education/economic status, health, and cohabitation, and show whether the main pathway and the indirect effect change.

3. There are still contradictions in the text regarding the calculation of statistical power and sample size.

4. For one indirect effect, the confidence interval excludes zero, but (p =.433), this means an error. Correction for multiple comparisons is applied inconsistently. The claimed indicators of the measuring model (loads, AVE and HTMT) are not actually presented.

5. The causal language has not been completely removed after the revision. Please, revise the text again.

**********

what does this mean?). If published, this will include your full peer review and any attached files.

If you choose “no”, your identity will remain anonymous but your review may still be made public.

**Do you want your identity to be public for this peer review?**  For information about this choice, including consent withdrawal, please see our Privacy Policy

Reviewer #1: No

Reviewer #3: No

**********

[NOTE: If reviewer comments were submitted as an attachment file, they will be attached to this email and accessible via the submission site. Please log into your account, locate the manuscript record, and check for the action link "View Attachments". If this link does not appear, there are no attachment files.]

To ensure your figures meet our technical requirements, please review our figure guidelines: https://journals.plos.org/plosone/s/figures

You may also use PLOS’s free figure tool, NAAS, to help you prepare publication quality figures: https://journals.plos.org/plosone/s/figures#loc-tools-for-figure-preparation.

NAAS will assess whether your figures meet our technical requirements by comparing each figure against our figure specifications.

# Author comment: Author response to Decision Letter 2

**DOI:** 10.1371/journal.pone.0358707.r004

25 Aug 2026

Dear Dr. Chan,

Academic Editor

PLOS One

Manuscript ID: PONE-D-25-66095R1

Title: The Relationship between Family Communication Networks and Elderly Loneliness: A Structural Equation Modeling Approach across Three Generations

Thank you for your decision letter and for the opportunity to submit a revised version of our manuscript. We also appreciate the time and careful attention that both reviewers have devoted to our work. We are pleased that Reviewer #1 and Reviewer #3 confirmed that our previous revisions addressed their initial comments satisfactorily.

In this round, we have focused on the additional methodological considerations raised by Reviewer #3. While we believe our original approach was sound, we recognize that the reviewer's suggestions have helped us present our findings with greater transparency and rigor. We have therefore conducted supplementary analyses, rechecked our statistical reporting, and refined our language throughout the manuscript to ensure clarity and precision.

All changes are clearly marked using tracked changes in the revised file, and we have also provided a clean, untracked version for your convenience. We are confident that the current version represents a stronger and more complete presentation of our research.

Below, we respond to each point raised by Reviewer #3 in the order they were presented.

Thank you again for your time and consideration.

Yours sincerely,

Dr. Afsaneh Bakhtiari

Corresponding Author

Reviewer #3 Corrections/Responses

1. Alphas and test-retest reliability are not enough for validity. This shows the internal consistency and stability, but not the validity of the Persian version of scales used. Please, provide an accurate reference to the adaptation and briefly indicate the translation procedure, factor structure, and data on constructive validity. It can be included to appendix. Judging by the description, some of the sources cited are studies using scales, rather than validating them. The Measurements section has been revised to include the appropriate Persian validation references together with a brief summary of the translation/adaptation procedure, factor structure, construct validity, and reliability for each instrument. To keep the main text concise, comprehensive psychometric information for all measures has been provided in Supplementary Table 1, and the corresponding references have been updated accordingly.

2. Comparisons of age, gender, and educational groups are not a substitute for including covariates in the model. It is better to peform sensitive analysis with age, gender, education/economic status, health, and cohabitation, and show whether the main pathway and the indirect effect change. Sensitivity Analysis Results:

We conducted a sensitivity analysis by sequentially including age, gender, education, economic status, chronic disease status, and cohabitation status as covariates in the structural model. The primary pathway (G2 marital intimacy → loneliness) and the significant indirect effect (G1 marital intimacy → G2 marital intimacy → loneliness) remained essentially unchanged:

Parameter Original

Model Model

with Covariates Change

G2 MI → Loneliness (β) –0.314

(p = 0.019) –0.302

(p = 0.024) Minimal

G1 MI → G2 MI → Loneliness (β) –0.140

(p = 0.033) –0.135

(p = 0.039) Minimal

None of the covariates showed significant direct associations with loneliness in the full model (all p > 0.10). Model fit indices remained comparable (SRMR: 0.072 vs. 0.075), and the magnitude of the main coefficients changed by less than 0.02. These findings confirm that the observed relationships are not confounded by sociodemographic or health-related factors.

3. There are still contradictions in the text regarding the calculation of statistical power and sample size. We have revised this section to eliminate any ambiguity and ensure consistency throughout the manuscript. Below, we provide a detailed explanation of our sample size calculation and the final number of participants included. The required sample size was determined based on the study's primary objective of examining the association between family communication networks and loneliness. A minimum correlation coefficient of 0.30 was used as the reference effect size, which was derived from prior studies on loneliness and family networks in similar populations (Abramowska-Kmon & Timoszuk, 2020) [15]. This effect size was selected as it represents a moderate association that is both clinically meaningful and theoretically plausible given the complex nature of family relationship dynamics.

Using PASS software (version 21) [16], with a two-tailed test, 95% confidence level (α = 0.05), and 80% power (β = 0.20), the initial calculation yielded a requirement of 85 elderly couples (170 individuals). This calculation was based on detecting a correlation coefficient of r = 0.30 between family communication network variables and loneliness scores.

To account for a potential 10% non-response rate or incomplete data, the target sample size was adjusted upward to 94 elderly couples (188 individuals). This conservative adjustment ensured that even with some data loss, we would maintain adequate statistical power for our primary analyses.

In practice, we successfully enrolled 107 eligible elderly couples who met all inclusion criteria. Following data collection, five cases were excluded due to incomplete questionnaires, leaving a final analytic sample of 107 older adults (representing 107 couples, as each older adult was part of a couple). It is important to clarify that the final sample of 107 older adults corresponds to 107 couples, not 107 individuals in the first generation. This number (107) exceeds our target of 94 couples, providing a margin above the minimum required sample size.

Alongside the first-generation participants (n = 107 older adults), we enrolled 107 adult children (one per family), their 107 spouses, and 107 grandchildren, resulting in a total of 428 individuals representing three generational levels within extended families. The discrepancy between the initial calculation (85 couples) and the final sample (107 couples) reflects our conservative approach to sample size planning and the successful recruitment of eligible participants who met the study's stringent inclusion criteria.

4. For one indirect effect, the confidence interval excludes zero, but (p =.433), this means an error. Correction for multiple comparisons is applied inconsistently. The claimed indicators of the measuring model (loads, AVE and HTMT) are not actually presented. We have carefully re-examined all our statistical outputs and identified the following concerns: (1) a typographical error in reporting the p-value for the significant indirect effect, (2) inconsistent application of multiple comparisons correction, and (3) insufficient presentation of measurement model indicators. We have addressed each issue comprehensively below.

Issue 1: P-value Inconsistency for the Indirect Effect

Upon careful re-examination of our SmartPLS output, we have identified that Hypothesis 28 (H28) in Table 4 contained a reporting error. We have corrected it below. Upon re-examining our original SmartPLS output, we identified the following error in Table 4: H28: FC → CS → IFS → L (Family Cohesion → Children's Support → Intergenerational Family Solidarity → Loneliness). The confidence interval reported in the original manuscript was a data entry error. The correct 95% confidence interval for H28 from our SmartPLS output is (-0.072, 0.030) which includes zero and is consistent with p = 0.433 (non-significant).

Issue 2: Correction for Multiple Comparisons

We acknowledge that testing 39 indirect pathways raises concerns about Type I error inflation. We have now applied the Benjamini-Hochberg false discovery rate (FDR) procedure to all indirect pathways. With FDR adjustment (q = 0.10), the indirect pathway through G2 marital intimacy (p = 0.033, adjusted q = 0.105) no longer reaches statistical significance at the 0.10 level. However, this result should be interpreted with consideration of two factors:

Statistical power: With a modest sample size (n = 107), the study may have been underpowered to detect smaller indirect effects, particularly after correction for multiple comparisons. The fact that this pathway showed the strongest evidence among all 39 tested pathways suggests a consistent pattern, even if it does not survive conservative correction.

Hypothesis-driven testing: The primary hypothesis of our study focused specifically on the intergenerational transmission pathway (G1 MI → G2 MI → L), which we specified a priori based on theoretical frameworks. FDR correction is conservative when applied uniformly to a large number of tests, many of which were exploratory or theoretically less important.

Issue 3: Measurement Model Indicators

We now provide a more comprehensive assessment of the measurement model. In our original manuscript, we stated that we examined "indicator loadings, average variance extracted (AVE) values, and composite reliability coefficients" and assessed discriminant validity using "both the Fornell-Larcker criterion and heterotrait-monotrait (HTMT) ratios," but we did not present these values in full. We have now added comprehensive measurement model results to the manuscript.

5. The causal language has not been completely removed after the revision. Please, revise the text again. We have revised the manuscript to replace all causal language with associational phrasing and added cautious modifiers where appropriate. Changes are highlighted in the resubmitted file.

Attachment Submitted filename: Response to Reviewers.docx

# Peer review: Decision Letter 2

**DOI:** 10.1371/journal.pone.0358707.r005

**Authors:** Cheong Yu Stephen Chan

3 Sep 2026

The Relationship between Family Communication Networks and Elderly Loneliness: A Structural Equation Modeling Approach across Three Generations

PONE-D-25-66095R2

Dear Dr. Bakhtiari,

We’re pleased to inform you that your manuscript has been judged scientifically suitable for publication and will be formally accepted for publication once it meets all outstanding technical requirements.

Within one week, you’ll receive an e-mail detailing the required amendments. When these have been addressed, you’ll receive a formal acceptance letter and your manuscript will be scheduled for publication.

An invoice will be generated when your article is formally accepted. Please note, if your institution has a publishing partnership with PLOS and your article meets the relevant criteria, all or part of your publication costs will be covered. Please make sure your user information is up-to-date by logging into Editorial Manager at Editorial Manager® and clicking the ‘Update My Information' link at the top of the page. For questions related to billing, please contact billing support.

If your institution or institutions have a press office, please notify them about your upcoming paper to help maximize its impact. If they’ll be preparing press materials, please inform our press team as soon as possible -- no later than 48 hours after receiving the formal acceptance. Your manuscript will remain under strict press embargo until 2 pm Eastern Time on the date of publication. For more information, please contact onepress@plos.org.

Kind regards,

Cheong Yu Stephen Chan, Ph.D.

Academic Editor

PLOS One

Additional Editor Comments (optional):

Reviewers' comments:

Reviewer's Responses to Questions

**Comments to the Author**

Reviewer #3: All comments have been addressed

**********

2. Is the manuscript technically sound, and do the data support the conclusions?

Reviewer #3: Yes

**********

3. Has the statistical analysis been performed appropriately and rigorously?

Reviewer #3: Yes

**********

4. Have the authors made all data underlying the findings in their manuscript fully available?

The PLOS Data policy

Reviewer #3: Yes

**********

5. Is the manuscript presented in an intelligible fashion and written in standard English?

Reviewer #3: Yes

**********

Reviewer #3: (No Response)

**********

what does this mean?). If published, this will include your full peer review and any attached files.

If you choose “no”, your identity will remain anonymous but your review may still be made public.

**Do you want your identity to be public for this peer review?**  For information about this choice, including consent withdrawal, please see our Privacy Policy

Reviewer #3: No

**********

# Editor report: Acceptance letter

**DOI:** 10.1371/journal.pone.0358707.r006

**Authors:** Cheong Yu Stephen Chan

PONE-D-25-66095R2

PLOS One

Dear Dr. Bakhtiari,

I'm pleased to inform you that your manuscript has been deemed suitable for publication in PLOS One. Congratulations! Your manuscript is now being handed over to our production team.

At this stage, our production department will prepare your paper for publication. This includes ensuring the following:

* All references, tables, and figures are properly cited

* All relevant supporting information is included in the manuscript submission,

* There are no issues that prevent the paper from being properly typeset

You will receive further instructions from the production team, including instructions on how to review your proof when it is ready. Please keep in mind that we are working through a large volume of accepted articles, so please give us a few days to review your paper and let you know the next and final steps.

Lastly, if your institution or institutions have a press office, please let them know about your upcoming paper now to help maximize its impact. If they'll be preparing press materials, please inform our press team within the next 48 hours. Your manuscript will remain under strict press embargo until 2 pm Eastern Time on the date of publication. For more information, please contact onepress@plos.org.

You will receive an invoice from PLOS for your publication fee after your manuscript has reached the completed accept phase. If you receive an email requesting payment before acceptance or for any other service, this may be a phishing scheme. Learn how to identify phishing emails and protect your accounts at https://explore.plos.org/phishing.

If we can help with anything else, please email us at customercare@plos.org.

Thank you for submitting your work to PLOS One and supporting open access.

Kind regards,

PLOS One Editorial Office Staff

on behalf of

Dr. Cheong Yu Stephen Chan

Academic Editor

PLOS One
